# Perianal Fistulae Medical Imaging Maps: A Pictorial Diagnostic Review

**DOI:** 10.3390/diagnostics16162584

**Published:** 2026-08-15

**Authors:** Sultan Abdulwadoud Alshoabi, Abdulkhaleq Ayedh Binnuhaid, Abdullatif O. Magram, Abdullgabbar M. Hamid, Fahad H. Alhazmi, Abdulkareem Algahtani, Bashair Alhummiany, Kamal D. Alsultan, Alaa Alshoabi, Rahaf Hamid, Awatif M. Omer, Mahmoud S. Babiker, Amel F. Alzain, Fathelrehman A. Elajab, Khaled Mohammed Al-Sayaghi

**Affiliations:** 1 Department of Diagnostic Radiology, College of Applied Medical Sciences, Taibah University, Al-Madinah 42353, Saudi Arabia; 2 Department of Specialized Surgery, Radiology Section, Faculty of Medicine, Hadhramout University, Hadhramaut P.O.B. 8892, Yemen; 3 Advanced AlRazi Diagnostic Center, Al-Hodeidah, Yemen; 4 Radiology Department, Rush University Medical Group, Chicago, IL 60612, USA; gabbaryy@gmail.com; 5 Applied College, Taibah University, Al-Madinah 42353, Saudi Arabia; 6 Biology Major, Dominican University, 7900 West Division Street, River Forest, IL 60305, USA; 7 Department of Medical Surgical Nursing, College of Nursing, Taibah University, Al-Madinah 42353, Saudi Arabia; 8 Nursing Division, Faculty of Medicine and Health Sciences, Sana’a University, Sana’a, Yemen

**Keywords:** perianal fistula, internal opening (IO), external opening (EO), fistulous tract, transcutaneous ultrasound imaging (TCUS), pelvic magnetic resonance imaging (MRI)

## Abstract

Perianal fistula, or anal fistula, is an abnormal granulomatous tract connecting the anal canal or rectum to the surrounding skin. It causes perianal pain and fistulous discharge, negatively impacting patients’ quality of life. The mechanism underlying fistula formation remains incompletely understood. Medical imaging is essential for accurate preoperative assessment of the shape, number and branching of fistulous tracts, identification of internal openings and Parks’ classification. This pictorial review provides an image-based overview of the key medical imaging concepts essential for the diagnosis of perianal fistulae and summarises the roles, advantages and limitations of the available imaging modalities for evaluating perianal fistulae. The current evidence on X-ray fistulography, computed tomography (CT) fistulography, transcutaneous ultrasonography (TCUS), endoanal ultrasonography (EAUS) and pelvic magnetic resonance imaging (MRI) was reviewed, emphasising on their diagnostic performance and clinical applications. Conventional X-ray fistulography has progressively been abandoned because it is invasive, is uncomfortable for patients, provides poor soft tissue contrast and inadequately visualises anal sphincters, complex fistulous tracts, secondary branches and abscesses. CT fistulography is useful for evaluating perianal abscesses and fistula when MRI and EAUS are unavailable. TCUS is widely available, non-invasive and radiation-free but is operator-dependent and has limited ability to evaluate high perianal fistulae. EAUS accurately evaluates fistulous tracts, can be performed at the bedside or intraoperatively and is reliable when MRI is contraindicated. However, EAUS is operator-dependent, is contraindicated in patients with severe anal stenosis or acute anal pain, and has a small field of view. Pelvic MRI is the gold standard, providing excellent visualisation of anal canal anatomy, superior soft tissue contrast, accurate mapping of fistulous tracts, localisation of internal openings, assessment of the involvement of anal sphincters and detection of secondary tracts, abscesses and associated complications. Consequently, MRI is a preferred imaging modality, which can guide treatment decisions and monitoring of treatment response.

## 1. Introduction

Perianal fistula, also known as an anal fistula, is an abnormal tract lined by granulomatous tissue that connects the anal canal or rectum to the surrounding perianal skin. It is a common anorectal condition, affecting approximately 12.3 men and 5.6 women per 100,000 population. The incidence peaks between 20 and 40 years of age [1]. Perianal fistulae can severely impact patients’ quality of life, causing considerable physical discomfort and psychological distress through symptoms such as perianal pain, fistulous discharge, restricted mobility and fatigue [2].

Clinical examination can confirm the presence of a perianal fistula. However, assessment may be challenging in patients with scarring or anatomical deformity, particularly when atypical primary tracts or secondary extensions are present. Careful examination under anesthesia therefore remains an important component of the overall assessment. Nevertheless, medical imaging is essential for accurate preoperative evaluation. It enables delineation of the number, extent, and branching patterns of fistulous tracts, identification of internal orifices, and classification according to the Parks system, which is fundamental to surgical planning and management [3].

The Parks classification categorizes perianal fistulae into four types according to the relationship of the fistulous tract to the internal anal sphincter (IAS) and the external anal sphincter (EAS). Intersphincteric fistulae are confined to the intersphincteric plane, whereas transsphincteric fistulae pass through the EAS. Suprasphincteric fistulae ascend within the intersphincteric plane above the puborectalis muscle before descending through the levator ani muscle into the ischiorectal fossa. Extrasphincteric fistulae lie entirely outside the EAS [4]. The American Society of Colon and Rectal Surgeons Standards Practice Task Force further categorizes perianal fistulae as either simple or complex. Simple fistulae include intersphincteric and low transsphincteric fistulae involving <30% of the EAS. Complex fistulae include high transsphincteric fistulae involving >30% of the EAS and >50% of the IAS, as well as suprasphincteric, extrasphincteric and horseshoe fistulae [5].

Imaging of the anal canal, rectum and surrounding soft tissues is technically challenging. Consequently, modalities such as magnetic resonance imaging (MRI) and endoanal ultrasonography (EAUS) play a pivotal role in the evaluation [6]. Pelvic MRI and EAUS are considered first-line imaging modalities because they can accurately delineate fistulous tracts, guide treatment planning, and improve surgical outcomes [7]. Given the central role of imaging in the management of perianal fistulae, there is a need for applying techniques that are accurate, widely available, cost-effective, and well tolerated by patients.

This review provides an image-based overview of the main imaging techniques relevant to the diagnosis of perianal fistulae. Although perianal fistulae have been studied extensively, relatively few comprehensive pictorial reviews directly compare the strengths and limitations of the available imaging modalities. This review is therefore intended to serve as a practical resource for radiologists, surgeons, and internal medicine physicians involved in the diagnosis and management of perianal fistulae.

## 2. Materials and Methods

This pictorial review focuses on the imaging features of perianal fistulae and discusses the key elements required for their comprehensive evaluation. These include localization of the internal and external openings, delineation of the primary fistulous tracts and secondary branches, identification of associated complications, and assessment of the tract’s relationship to the internal and external anal sphincters.

Characteristic radiological findings identified in the literature were illustrated using representative cases retrieved retrospectively from the imaging archive of two diagnostic centers: Alsafwa Consultative Medical Center (ACMC), Almukalla, Hadhramout, Republic of Yemen, and Advanced AlRazi Diagnostic Center, Al-Hodeidah, Republic of Yemen. Selected fistulography, transcutaneous ultrasonography (TCUS), and pelvic MRI images demonstrating classical imaging features of perianal fistulae were included. All images were independently evaluated by two experienced radiologists to ensure diagnostic accuracy and optimal image quality. Images were selected based on their ability to clearly demonstrate the key diagnostic features, including IOs, primary fistulous tracts, secondary branches, abscess formation, and EOs.

**Figure 1:**
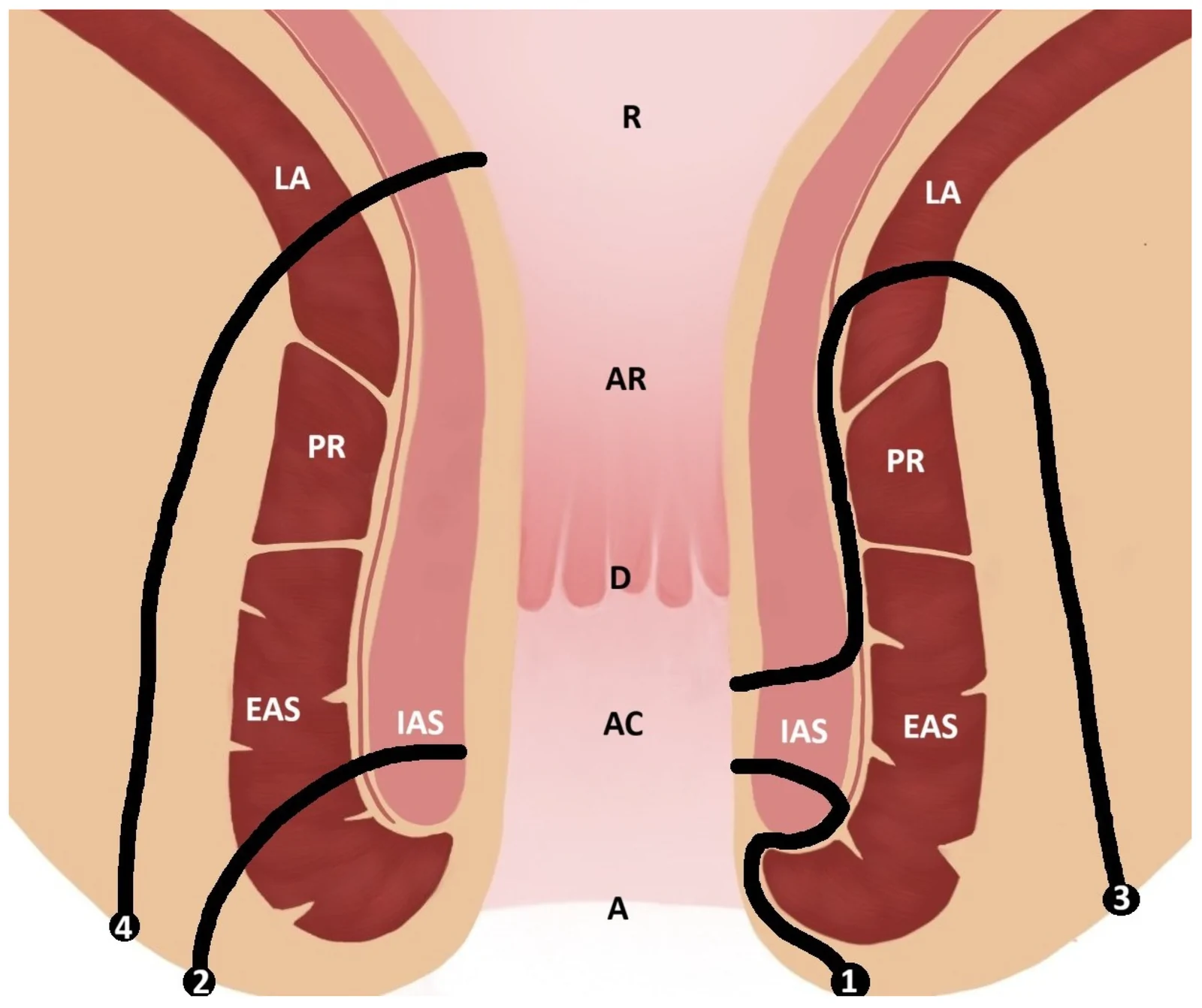
The diagram illustrates the anal and perianal anatomy, showing the anal verge (A), anal canal (AC), dentate line (D), anorectal junction (AR), rectum (R), internal anal sphincter (IAS), external anal sphincter (EAS), puborectalis muscle (PR) and levator ani muscle (LA). The diagram also shows the four types of perianal fistulae: intersphincteric fistula (1), transsphincteric fistula (2), suprasphincteric fistula (3), and extrasphincteric fistula (4).

A targeted literature review was conducted to identify relevant studies on the anatomy, pathophysiology, and imaging features of perianal fistulae, as well as their diagnostic significance. The evidence was synthesized to compare the available imaging modalities and describe their respective advantages and limitations. Particular attention was given to TCUS and pelvic MRI, with emphasis on the potential role of TCUS as a readily available, non-invasive, and radiation-free imaging modality. The Sketchbook, online, (https://www.sketchbook.com/) was used for drawing Figure 1 of this article.

## 3. Anatomy of the Anal and Perianal Region

The anal canal is the final 4 cm segment of the gastrointestinal tract, extending from the anorectal ring to the anal verge. Its primary functions are to maintain fecal continence and facilitate defecation. It consists of a complex cylindrical double-layered muscular arrangement. The inner layer comprises the IAS and the conjoined longitudinal muscle (CLM), which are innervated by the autonomic nervous system. The outer layer consists of the thick EAS and the puborectalis muscle, which are innervated by the somatic nervous system. The EAS extends more distally than the IAS and constitutes the distal end of the anal canal while surrounding the IAS from outside to inside. The dentate (pectinate) line is a wavy line that divides the anal canal into an upper, pain-insensitive part innervated by the autonomic nervous system and a lower part, known as the anoderm, which consists of nonkeratinized squamous epithelium without glands. The anoderm is sensitive to pain as it is innervated by somatic nerves. The anal columns (columns of Morgagni) terminate at the dentate line, where the anal crypts are located. The anal crypts are connected by the anal gland ducts to the underlying anal glands. Obstruction of these ducts is considered the primary cause of the development of anal abscesses and fistulae [8,9,10]. (Figure 1).

The major muscles of the anal canal include the following:

1. IAS: It is a continuation of the inner circular smooth muscle of the rectum. It starts from the pelvic diaphragm and terminates approximately 1 cm proximal to the distal end of the EAS. The IAS surrounds the upper two-thirds of the anal canal and keeps the canal closed to prevent the leakage of gas and liquid stool.
2. CLM: It is a layer of muscular tissue interposed between the IAS and the EAS within the intersphincteric space, which contains a thin layer of fatty tissue.
3. EAS: It is a voluntary striated muscle that envelops the entire IAS and CLM. It receives somatic innervation via the pudendal nerve, providing conscious control of defecation and offering squeeze pressure during sudden increases in intra-abdominal pressure.
4. Puborectalis muscle: It is part of the levator ani muscle complex, which forms a muscular sling that pulls the rectum forwards, creating the anorectal angle. It plays a vital role in maintaining continence, and voluntary relaxation of this muscle straightens the anorectal angle to allow defecation.

**Figure 2:**
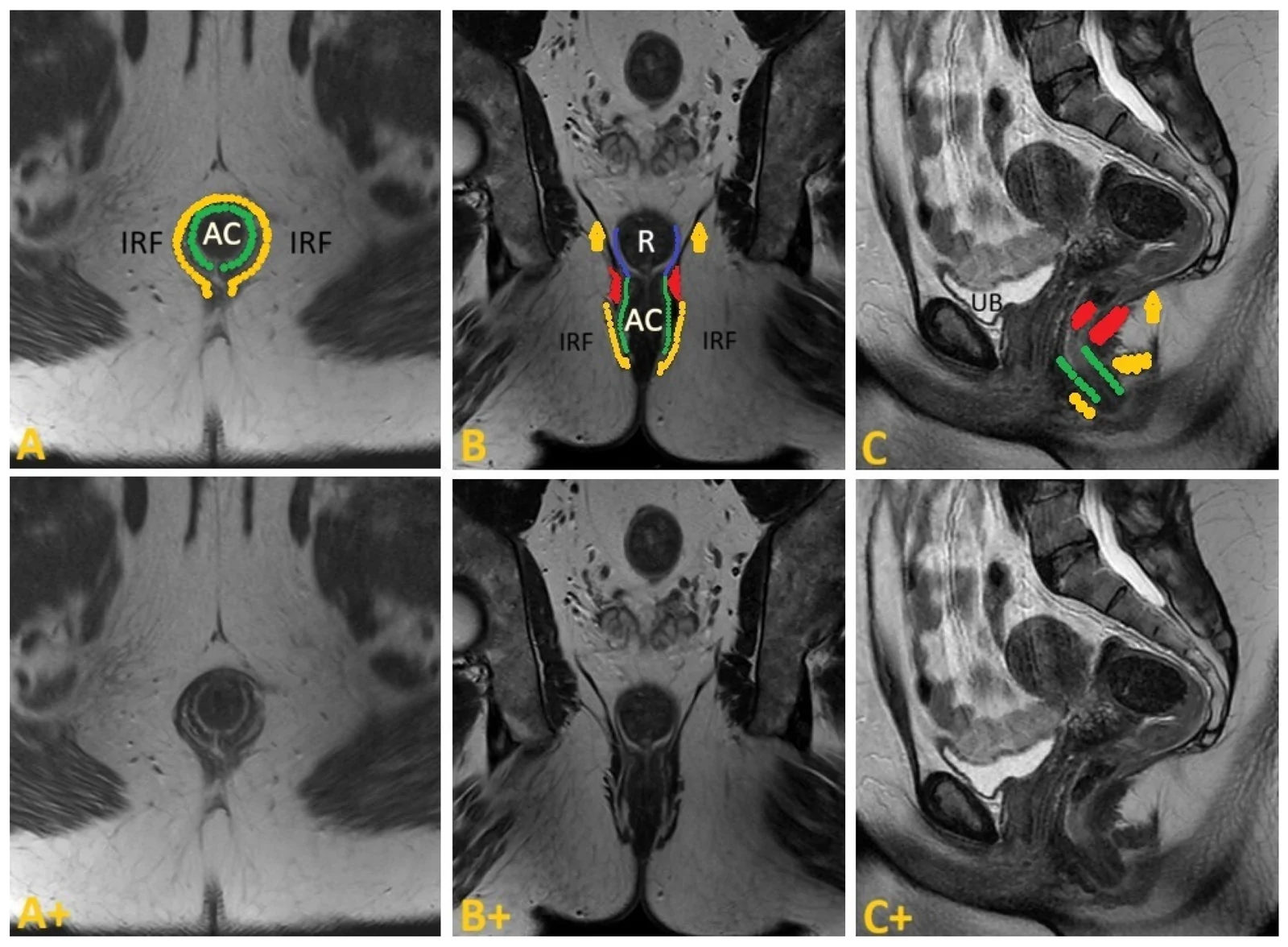
Pelvic MRI acquired using a 1.5 Tesla MRI machine with high resolution illustrating the normal anatomy of the anal canal. (**A**) Axial T2-weighted image showing the high signal intensity fat around the anal canal (AC) and bilateral ischiorectal fossae (IRF). (**A+**) Corresponding unannotated image. (**B**) Coronal T2-weighted image showing the internal anal sphincter (green line) continuous with the inner circular smooth muscle of the rectum (blue line), external anal sphincter (yellow line), the intersphincteric space in between, the puborectalis muscle (red lines), and the levator ani muscles bilaterally (arrows). (**B+**) Corresponding unannotated image. (**C**) Sagittal T2-weighted image showing the internal anal sphincter (green line), external anal sphincter (dotted yellow line), the puborectalis muscle (red lines), and the levator ani muscle (arrow). (**C+**) Corresponding unannotated image. UB: Urinary bladder.

**Figure 3:**
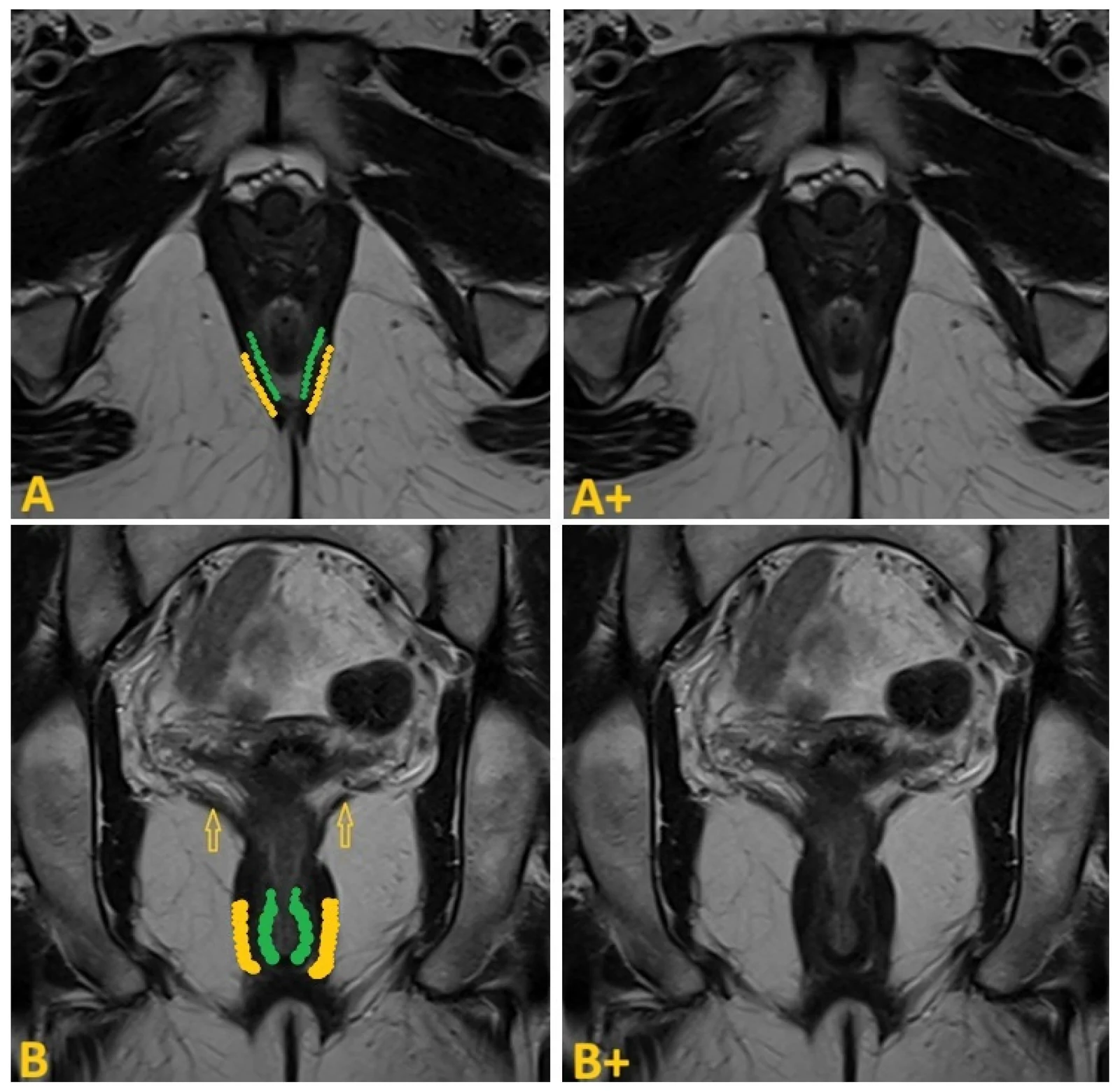
Pelvic MRI acquired using a 1.5 Tesla MRI machine with high resolution illustrating the normal anatomy of the anal canal. (**A**) Axial T2-weighted image showing the internal anal sphincter (green line), external anal sphincter (yellow line), the high-signal intersphincteric space between the sphincters, and the ischiorectal fossae (IRF). (**A+**) Corresponding unannotated image. (**B**) Coronal T2-weighted image showing the internal anal sphincter (green line), external anal sphincter (yellow line), the intersphincteric space, and the levator ani muscles bilaterally (arrows). (**B+**) Corresponding unannotated image.

MRI provides a precise structural and functional assessment of the pelvic compartments [11]. A thorough understanding of the MRI anatomy of the anal canal and perianal region enables systematic interpretation of fistulous disease and comprehensive assessment of its extent. MRI clearly demonstrates the IAS, EAS, puborectalis muscle, levator ani muscles, intersphincteric space, and ischiorectal fossae. In addition, it provides accurate delineation of fistulous anatomy, assessment of disease activity, and detection of associated complications [12] (Figure 2 and Figure 3).

## 4. Pathophysiology of Perianal Fistula

The mechanism underlying perianal fistula formation is not yet fully understood. Although the cause is often idiopathic, several predisposing conditions have been identified, including male sex, obesity, Crohn’s disease, pelvic infection, tuberculosis, childbirth-related trauma, diverticulitis, pelvic malignancies, and radiotherapy. The cryptoglandular hypothesis of pathogenesis suggests that infection of an anal gland within the intersphincteric space leads to the formation of an intersphincteric abscess, which may subsequently lead to the formation of a perianal fistula [13,14]. Perianal fistulae can be classified into two categories: (1) primary fistulae, which result from obstruction of the anal gland ducts, leading to stasis, abscess formation, and subsequent fistula development; and (2) secondary fistulae, which are associated with inflammatory bowel disease, malignancies, infections, or iatrogenic factors. Two primary mechanisms have been proposed to explain the pathogenesis of perianal fistulae. The first is epithelial-to-mesenchymal transition (EMT), a process in which specialized epithelial cells transform into mesenchymal cells, which can migrate and infiltrate adjacent tissues. EMT occurs during wound healing, embryonic development, organ formation, tumor growth, or metastasis. The second mechanism involves matrix metalloproteinases (MMPs), a family of enzymes that degrade components of the extracellular matrix. Increased MMP activity has been observed in patients with inflammatory bowel disease [1].

## 5. Medical Imaging of Perianal Fistula

Medical imaging is essential for managing perianal fistulae through accurate mapping of fistulous tracts and their branches, identifying hidden abscesses, and determining the relationship of the fistulous tract to the IAS and EAS. Precise visualization of the fistulous tract is critical for guiding surgery, preventing fecal incontinence, and reducing the recurrence rate. X-ray fistulography, CT fistulography, EAUS, and MRI are used to assess perianal fistulae. Among these, MRI is the preferred imaging modality [15]. The American College of Radiology Appropriateness Criteria designate pelvic MRI with or without contrast and contrast-enhanced pelvic CT as usually appropriate for the initial imaging of perianal abscesses or fistulae [16]. However, each imaging modality has its own advantages and limitations, which are discussed in the following sections.

### 5.1. X-Ray Fistulography

**Figure 4:**
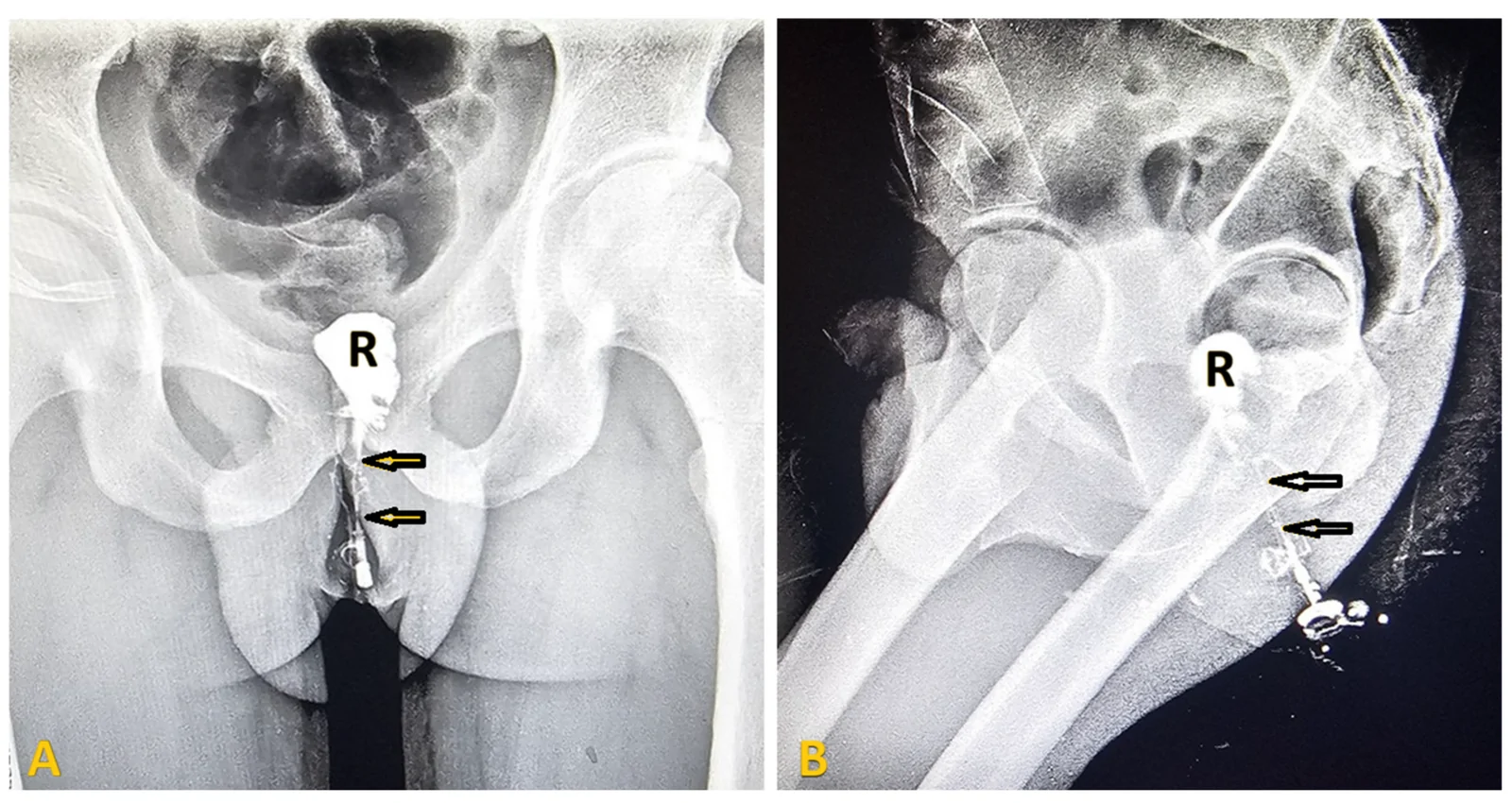
Selected X-ray fistulogram showing a high perianal fistula showing the contrast-filled fistulous tract (arrows) and contrast leakage into the rectum (R) on (**A**) anteroposterior and (**B**) lateral views.

X-ray fistulography is performed by instilling a radiopaque contrast medium through the EO, or through the IO in blind fistulae, into the fistulous tract, followed by radiographic imaging to delineate the anatomy of the fistula [1,17]. The clinical value of X-ray fistulography has progressively declined because of several limitations, including its invasive nature, poor soft tissue contrast, and inability to visualize the anal sphincters and their relationship to the fistula. It also has a high rate of missed or false tracts and provides suboptimal assessment of complex fistulae because tract branches and abscesses may not be properly filled with contrast. In addition, the procedure poses a risk of contrast-related complications and may be painful for patients [18] (Figure 4).

### 5.2. CT Fistulography

CT is primarily used in emergency or acute cases to evaluate or guide the drainage of large perianal abscesses. CT fistulography with contrast injection into the fistulous tract can help identify the course of the tract but requires experienced radiologists for both image acquisition and interpretation. Compared with MRI, CT has limited soft tissue resolution and exposes patients to ionizing radiation. Therefore, it is mainly limited to selected preoperative cases of complex perianal fistulae with multiple EOs or as a problem-solving modality in patients with contraindications to MRI [19].

### 5.3. TCUS

**Figure 5:**
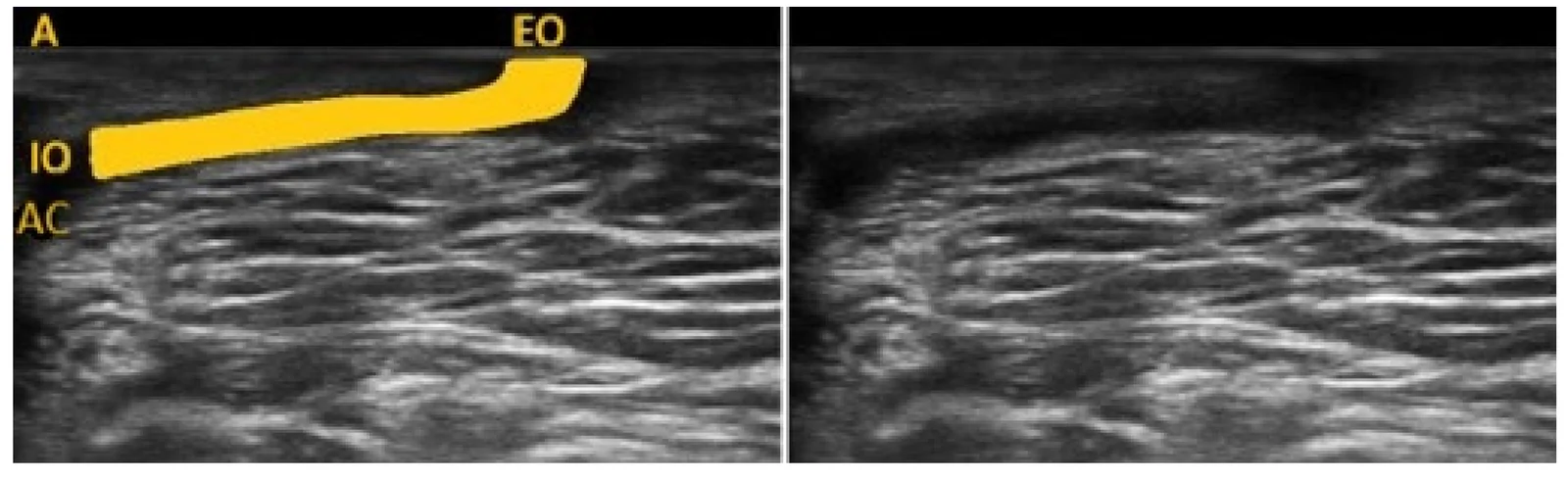
An ultrasound image obtained using a superficial linear probe (7.5 MHz) shows a simple low-type perianal fistula with a shallow fistulous tract measuring 4 cm in length, with an obvious external opening (EO) and an internal opening (IO) into the anal canal (AC) located 9 mm from the anal verge (A). No fistulous branching or abscess. The **left image** shows the tract of the fistula (thick yellow line), EO, and IO. The **right image** is the corresponding unannotated scan.

**Figure 6:**
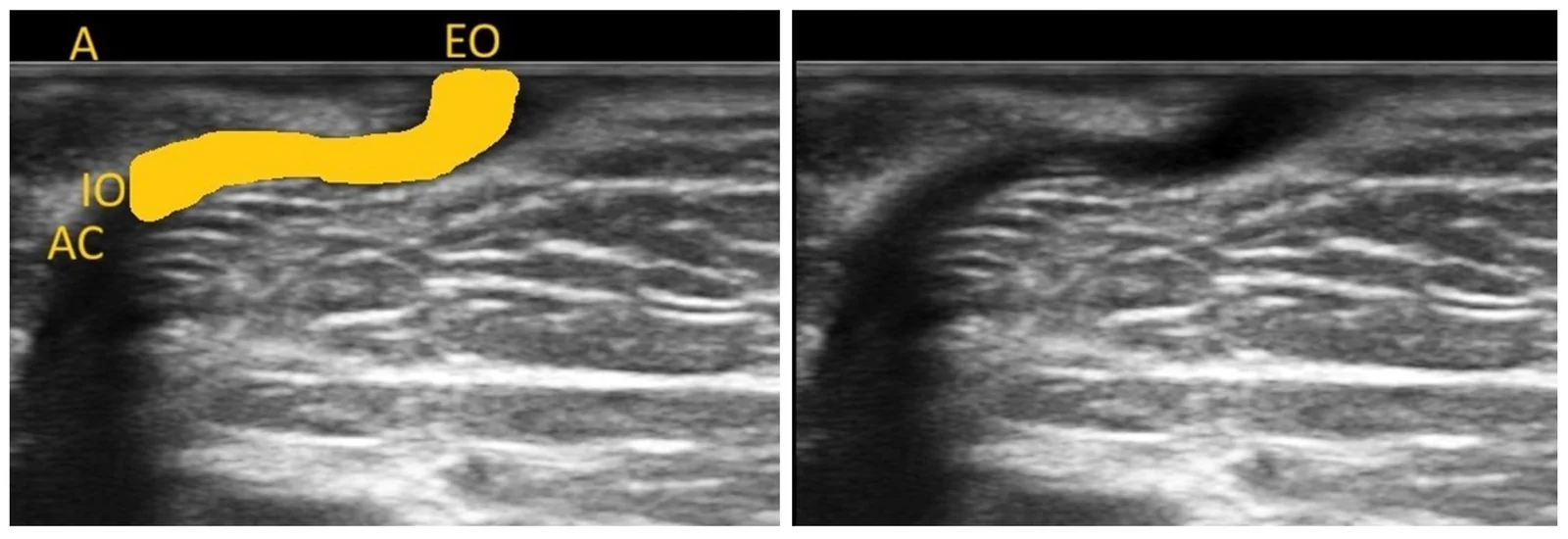
An ultrasound image obtained with a superficial linear probe (7.5 MHz) shows a simple low-type perianal fistula with a shallow fistulous tract measuring 3 cm in length, with an obvious external opening (EO) and an internal opening (IO) into the anal canal (AC) located 12 mm distant from the anal verge (A). No fistulous branching or abscess. The **left image** shows the tract of the fistula (thick yellow line), EO, and IO. The **right image** is the corresponding unannotated scan.

**Figure 7:**
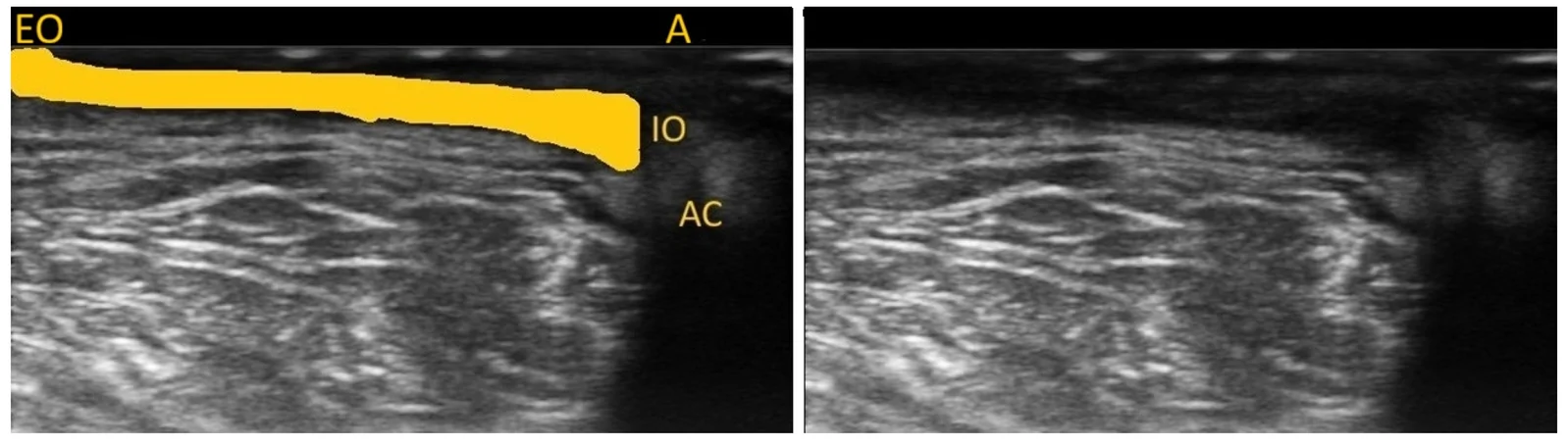
An ultrasound image obtained with a superficial linear probe (7.5 MHz) shows a simple low-type perianal fistula with a single fistulous tract measuring 5 cm in length, with an obvious external opening (EO) and an internal opening (IO) into the anal canal (AC) located 9 mm distant from the anal verge (A). No fistulous branching or abscess. The **left image** shows the tract of the fistula (thick yellow line), EO, and IO. The **right image** is the corresponding unannotated scan.

**Figure 8:**
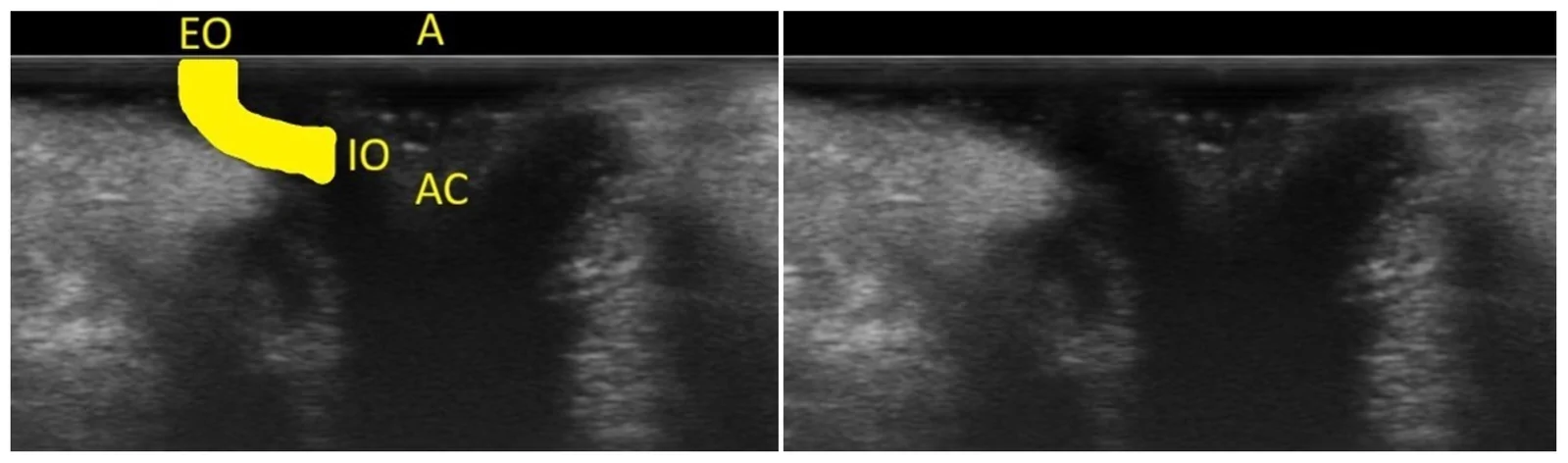
An ultrasound image obtained with a superficial linear probe (7.5 MHz) shows a simple low-type perianal fistula with a single fistulous tract measuring 1.5 cm in length, with an obvious external opening (EO) and an internal opening (IO) into the anal canal (AC) located 9 mm distant from the anal verge (A). No fistulous branching or abscess. The **left image** shows the tract of the fistula (thick yellow line), EO, and IO. The **right image** is the corresponding unannotated scan.

**Figure 9:**
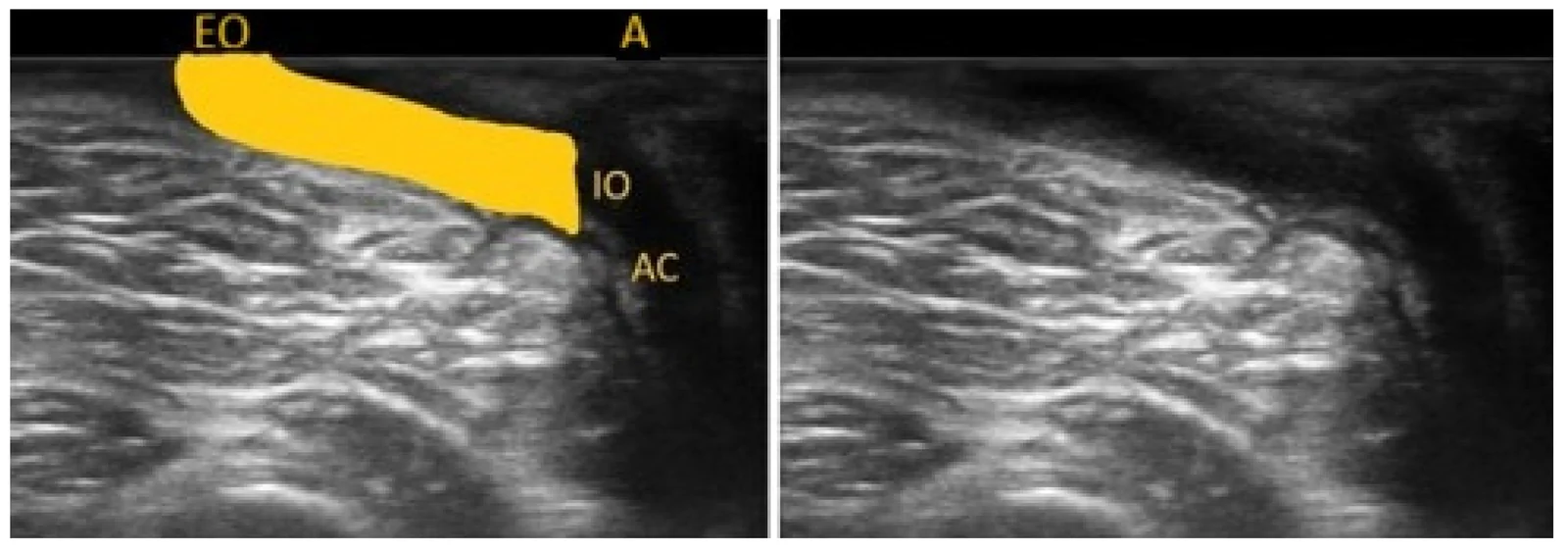
An ultrasound image obtained with a superficial linear probe (7.5 MHz) shows a simple low-type perianal fistula with a single fistulous tract measuring 3 cm in length, with an obvious external opening (EO) and an internal opening (IO) into the anal canal (AC) located 14 mm away from the anal verge (A). No fistulous branching or abscess. The **left image** shows the tract of the fistula (thick yellow line), EO, and IO. The **right image** is the corresponding unannotated scan.

**Figure 10:**
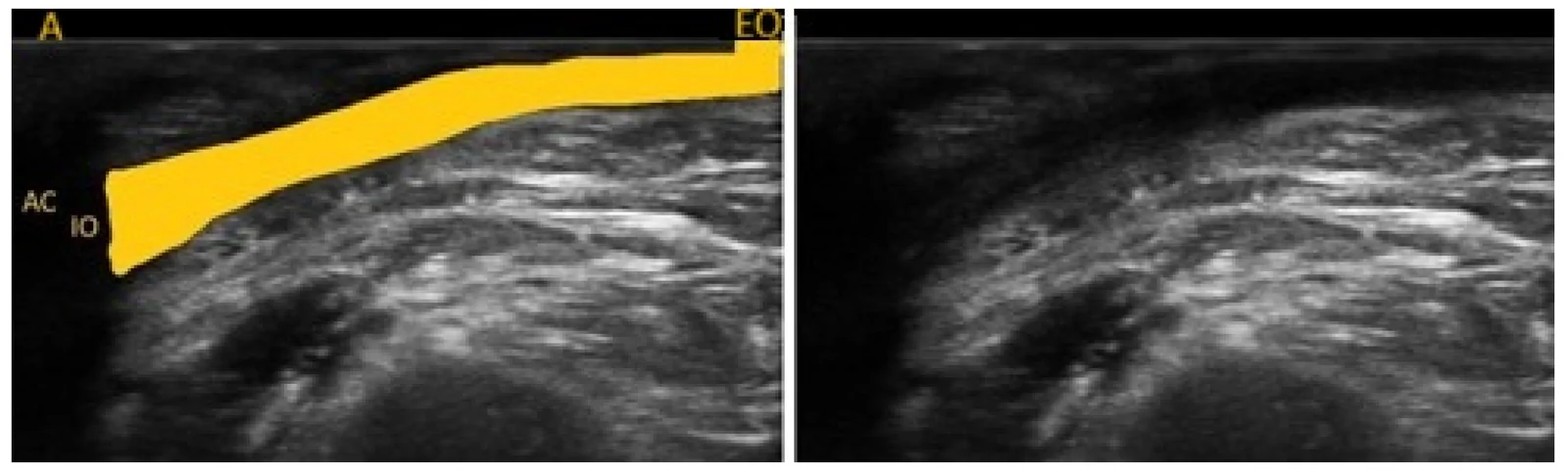
An ultrasound image obtained with a superficial linear probe (7.5 MHz) shows a simple linear low-type perianal fistula with a fistulous tract measuring 4 cm in length, with an obvious external opening (EO) and a single internal opening (IO) into the anal canal (AC) located 12 mm away from the anal verge (A). No fistulous branching or abscess. The **left image** shows the tract of the fistula (thick yellow line), EO, and IO. The **right image** is the corresponding unannotated image.

**Figure 11:**
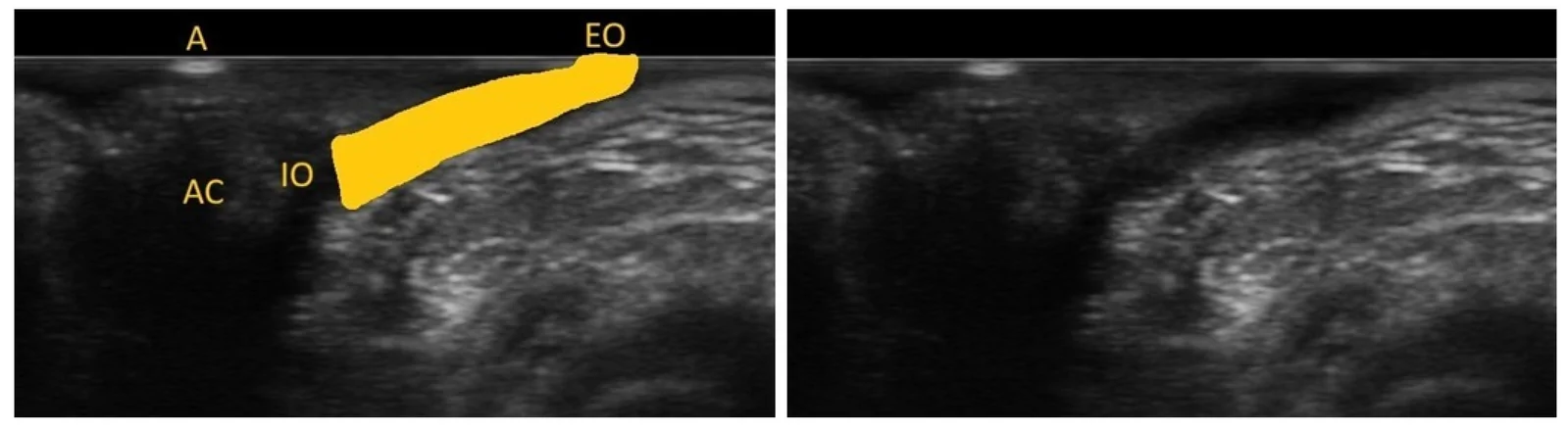
An ultrasound image obtained with a superficial linear probe (7.5 MHz) shows a simple linear low-type perianal fistula with a fistulous tract measuring 2 cm in length, with an obvious external opening (EO) and a single internal opening (IO) into the anal canal (AC) located 8 mm away from the anal verge (A). No fistulous branching or abscess. The **left image** shows the tract of the fistula (thick yellow line), EO, and IO. The **right image** is the corresponding unannotated scan.

**Figure 12:**
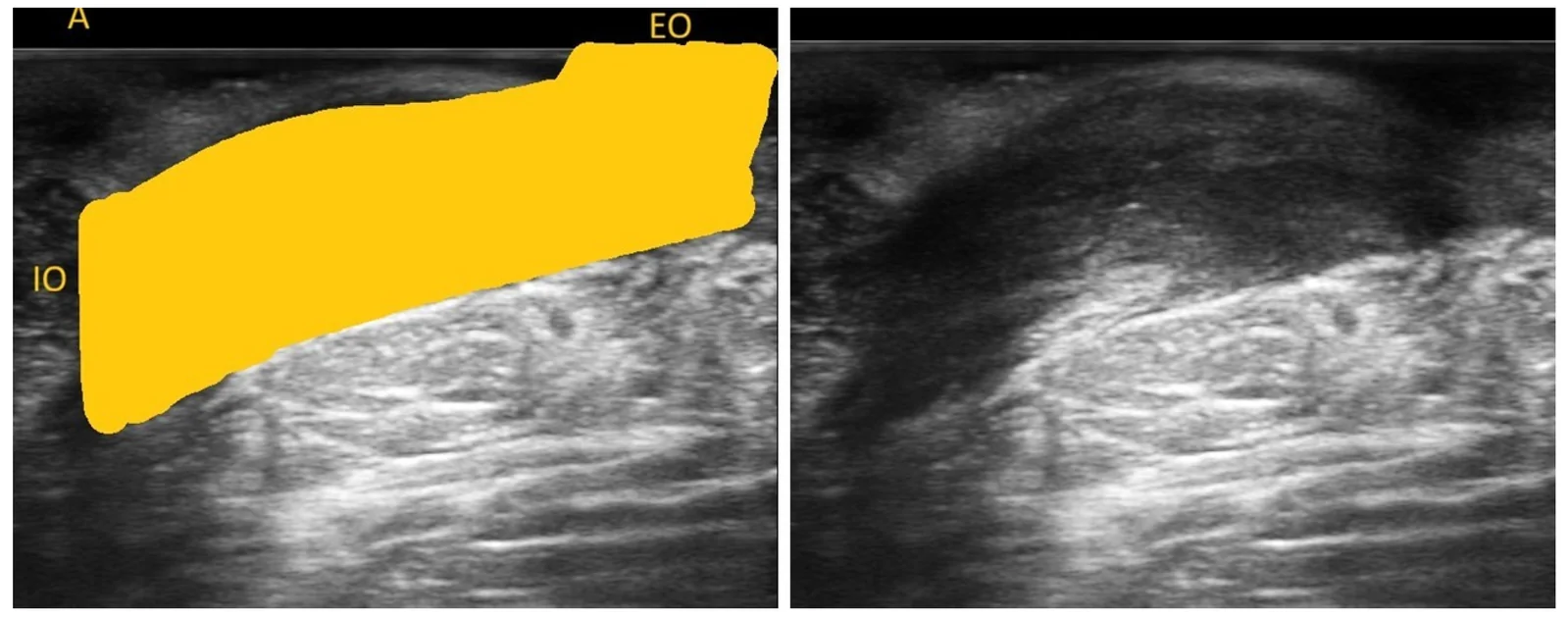
An ultrasound image obtained with a superficial linear probe (7.5 MHz) shows a simple low-type perianal fistula with a fistulous tract measuring 5 cm in length, with an obvious external opening (EO) and a single internal opening (IO) into the anal canal (AC) located 14 mm away from the anal verge (A). No fistulous branching or abscess. The **left image** shows the tract of the fistula (thick yellow line), EO, and IO. The **right image** is the corresponding unannotated scan.

**Figure 13:**
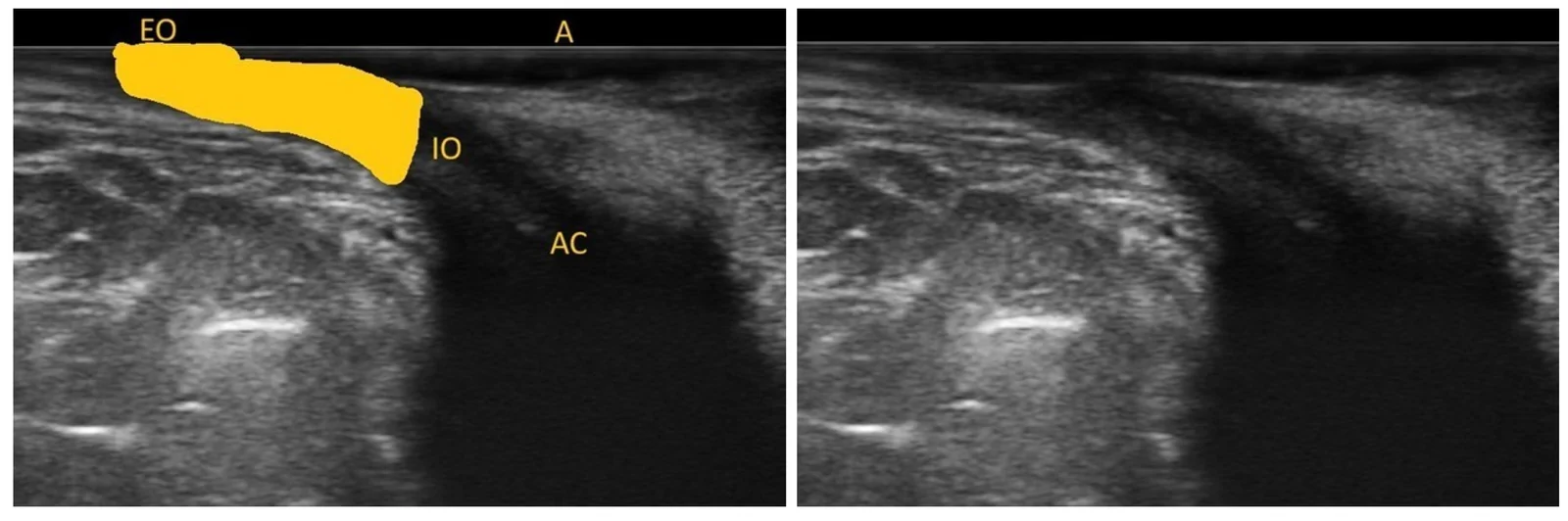
An ultrasound image obtained with a superficial linear probe (7.5 MHz) shows a simple low-type perianal fistula with a fistulous tract measuring 2.5 cm in length, with an obvious external opening (EO) and a single internal opening (IO) into the anal canal (AC) located 14 mm away from the anal verge (A). No fistulous branching or abscess. The **left image** shows the tract of the fistula (thick yellow line), EO, and IO. The **right image** is the corresponding unannotated scan.

**Figure 14:**
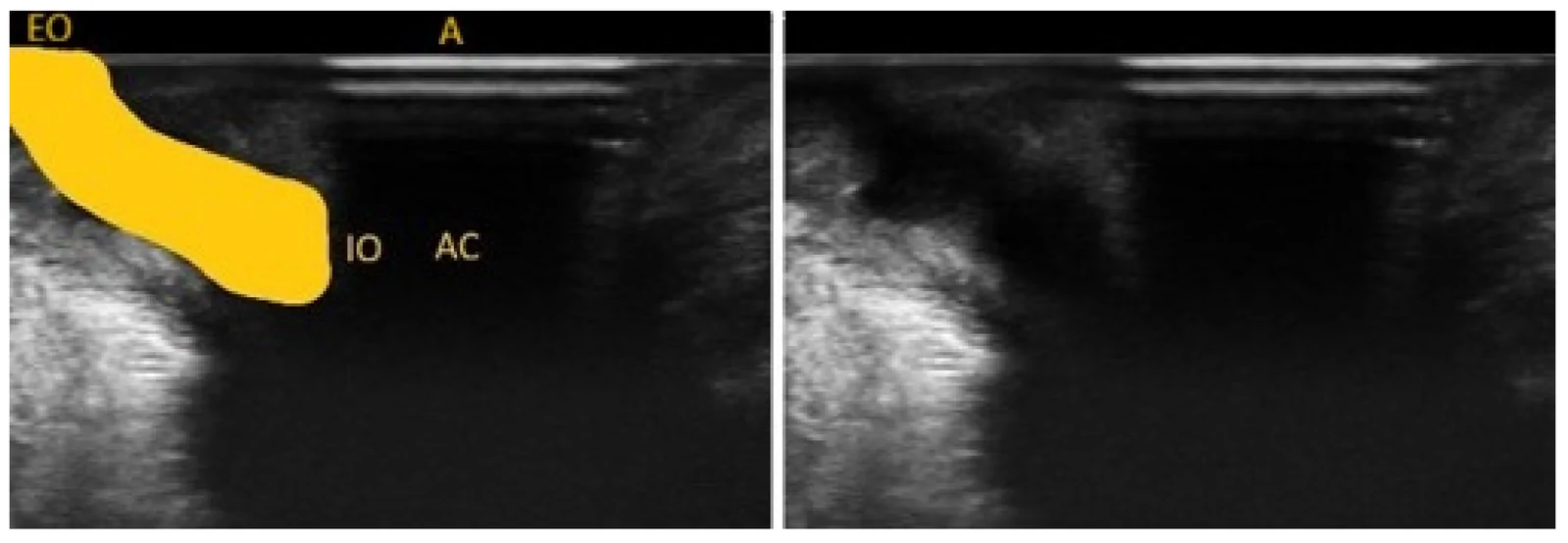
An ultrasound image obtained with a superficial linear probe (7.5 MHz) shows a low-type perianal fistula with a fistulous tract measuring 3 cm in length, with an obvious external opening (EO) and a single internal opening (IO) into the anal canal (AC) located 15 mm away from the anal verge (A). No fistulous branching or abscess. The **left image** shows the tract of the fistula (thick yellow line), EO, and IO. The **right image** is the corresponding unannotated scan.

**Figure 15:**
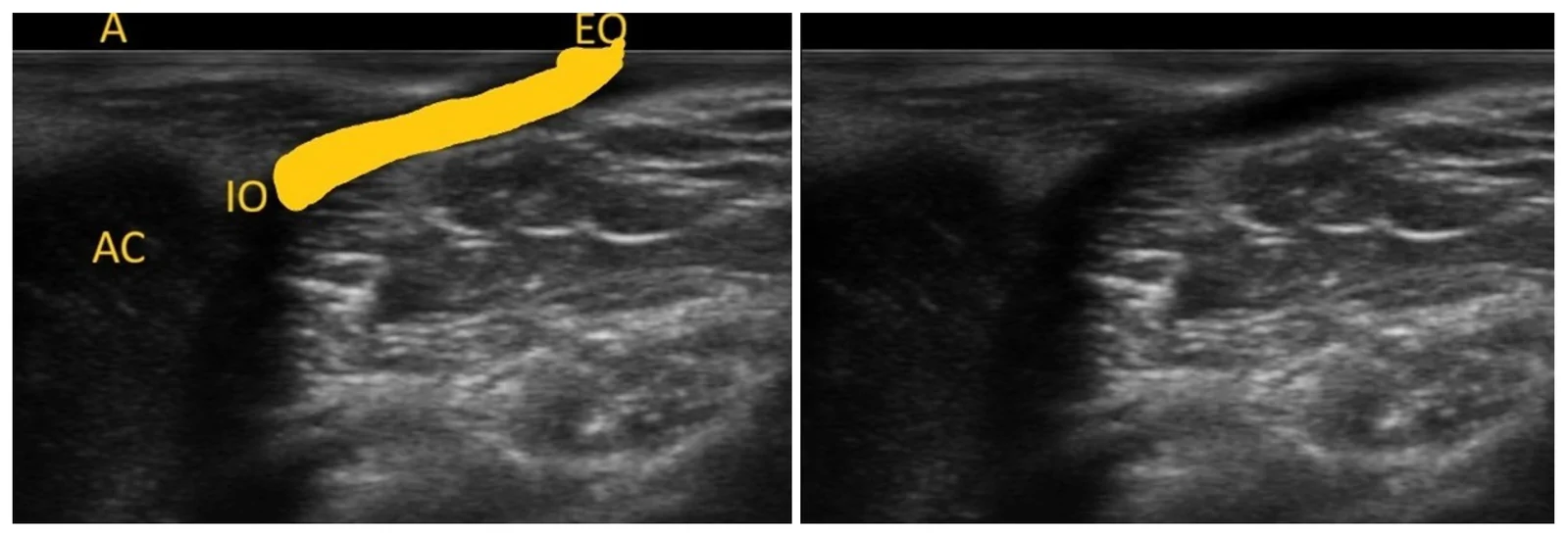
An ultrasound image obtained with a superficial linear probe (7.5 MHz) shows a low-type perianal fistula with a fistulous tract measuring 3 cm in length, with an obvious external opening (EO) and a single internal opening (IO) into the anal canal (AC) located 10 mm away from the anal verge (A). No fistulous branching or abscess. The **left image** shows the tract of the fistula (thick yellow line), EO, and IO. The **right image** is the corresponding unannotated scan.

**Figure 16:**
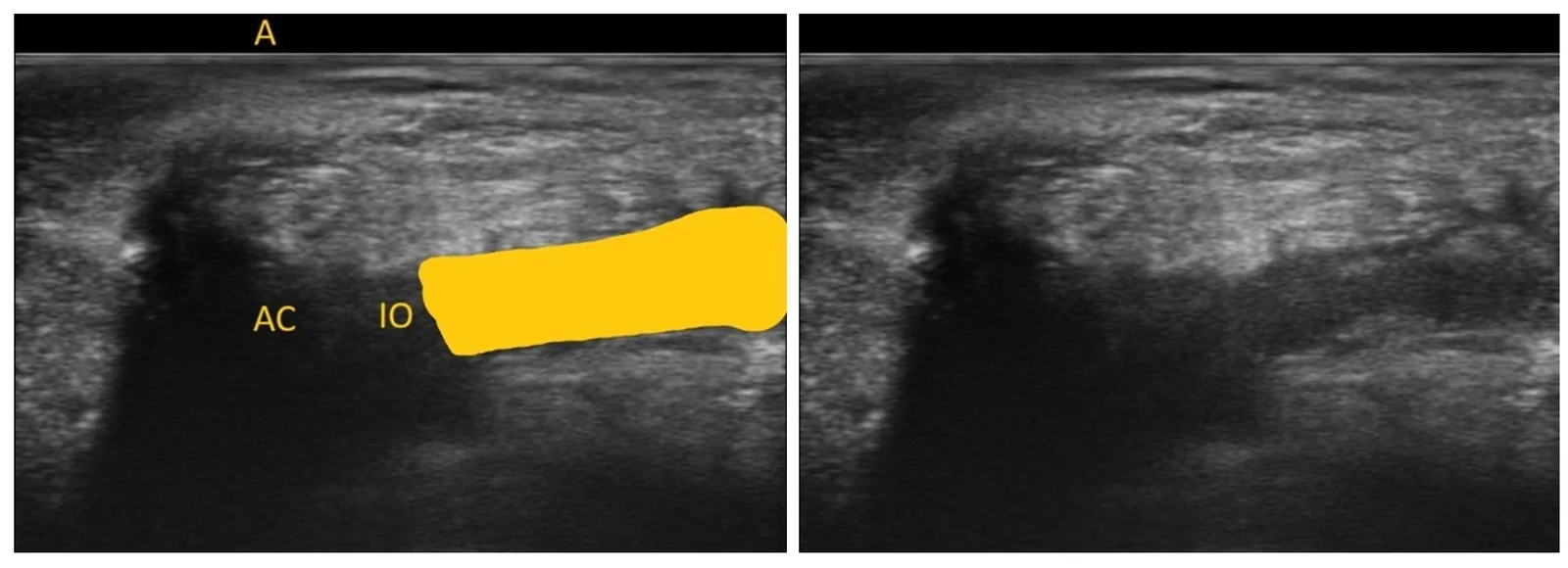
An ultrasound image obtained with a superficial linear probe (7.5 MHz) shows a low-type transsphincteric perianal fistula with a fistulous tract measuring 7 cm that could not be seen on the same image, with an obvious internal opening (IO) into the anal canal (AC) located 19 mm away from the anal verge (A). No fistulous branching or abscess. Magnetic resonance imaging was recommended for this patient for definitive Park classification. The **left image** shows the tract of the fistula (thick yellow line) and IO. The **right image** is the corresponding unannotated scan.

**Figure 17:**
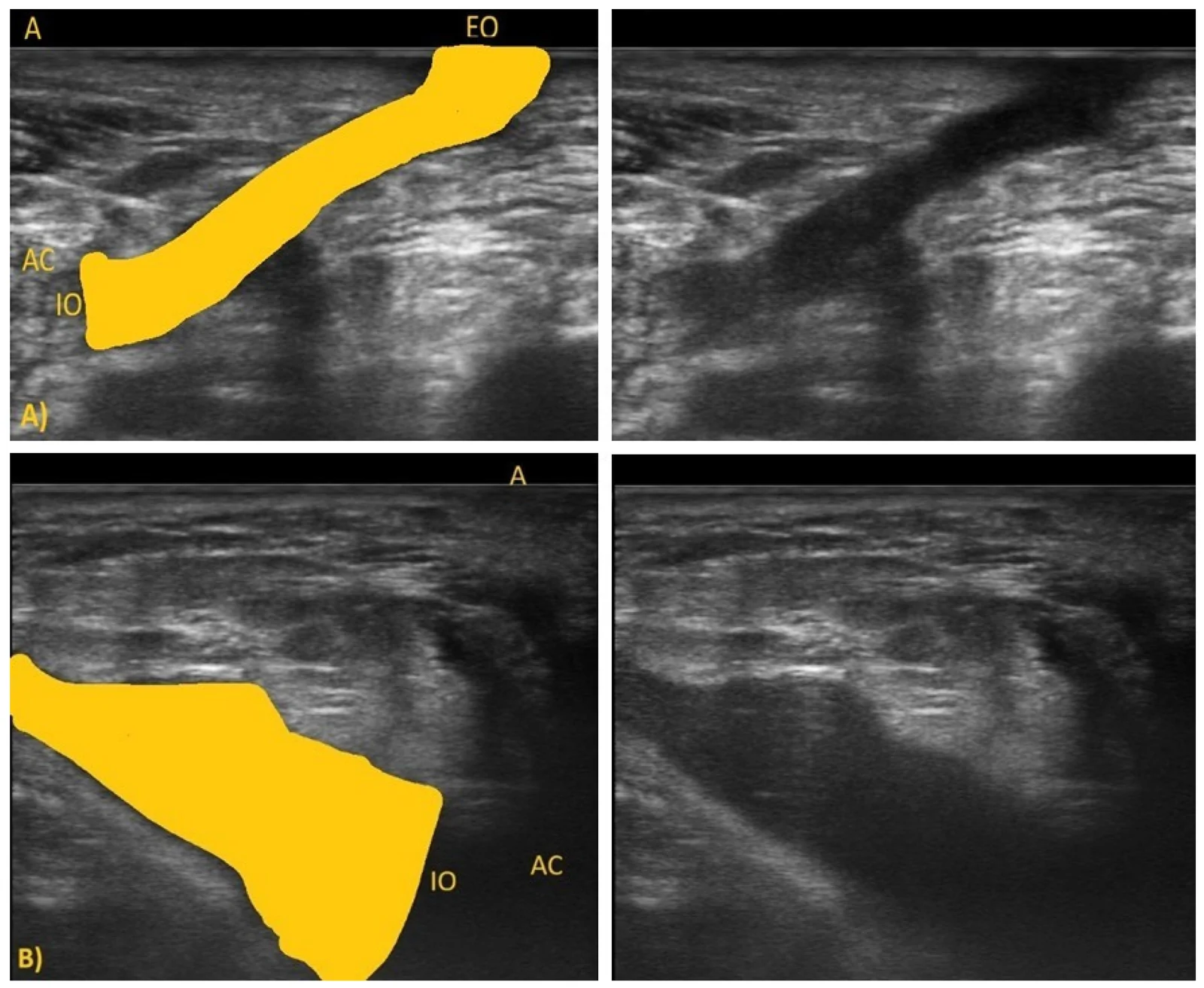
Ultrasound images obtained with a superficial linear probe (7.5 MHz) shows a complex bilateral perianal fistula with image (**A**) a right fistulous tract measuring 4 cm in length, with an obvious external opening (EO) and a single internal opening (IO) into the anal canal (AC) located 15 mm away from the anal verge (A), and image (**B**) Another left-sided fistulous tract with an IO into the AC located 34 mm away from the anal verge (A) with no EO. The **left images** show the tract of the fistula (thick yellow line), EO, and IO. The **right images** are the corresponding unannotated scans.

**Figure 18:**
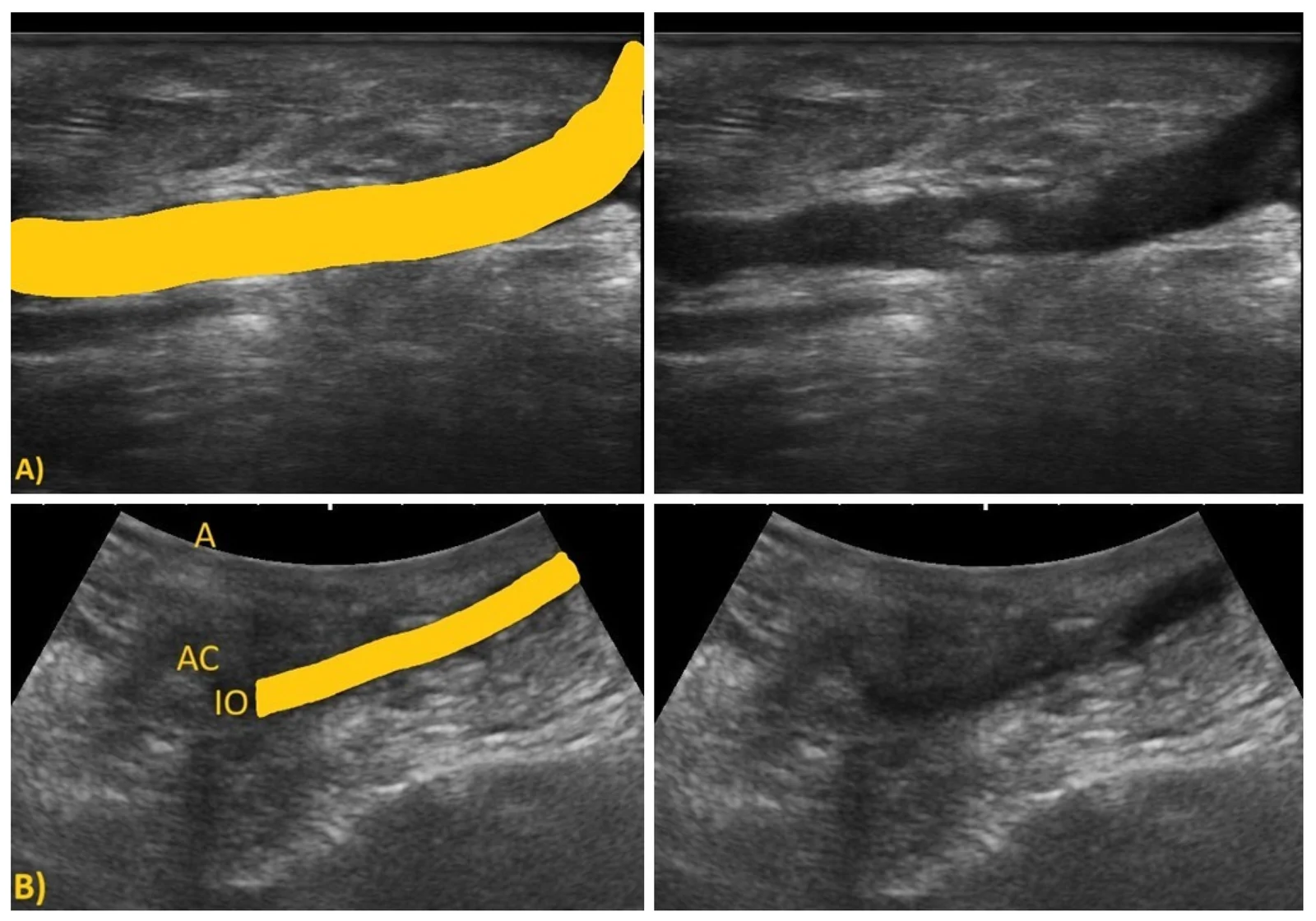
Ultrasound images: (**A**) Images obtained using a superficial linear probe (7.5 MHz) show a long, low-type fistulous tract, making it difficult to visualize the external opening (EO) and internal opening (IO) on the same scan. (**B**) Images obtained using a deep curved probe (3.5 MHz) show a 7 cm long fistulous tract with an obvious internal opening (IO) into the anal canal (AC). However, the external opening was difficult to visualize on the same scan due to the length of the fistula. The **left images** show the fistulous tract (thick yellow line) and the IO. The **right images** are the corresponding unannotated scans. A: Anal verge.

**Figure 19:**
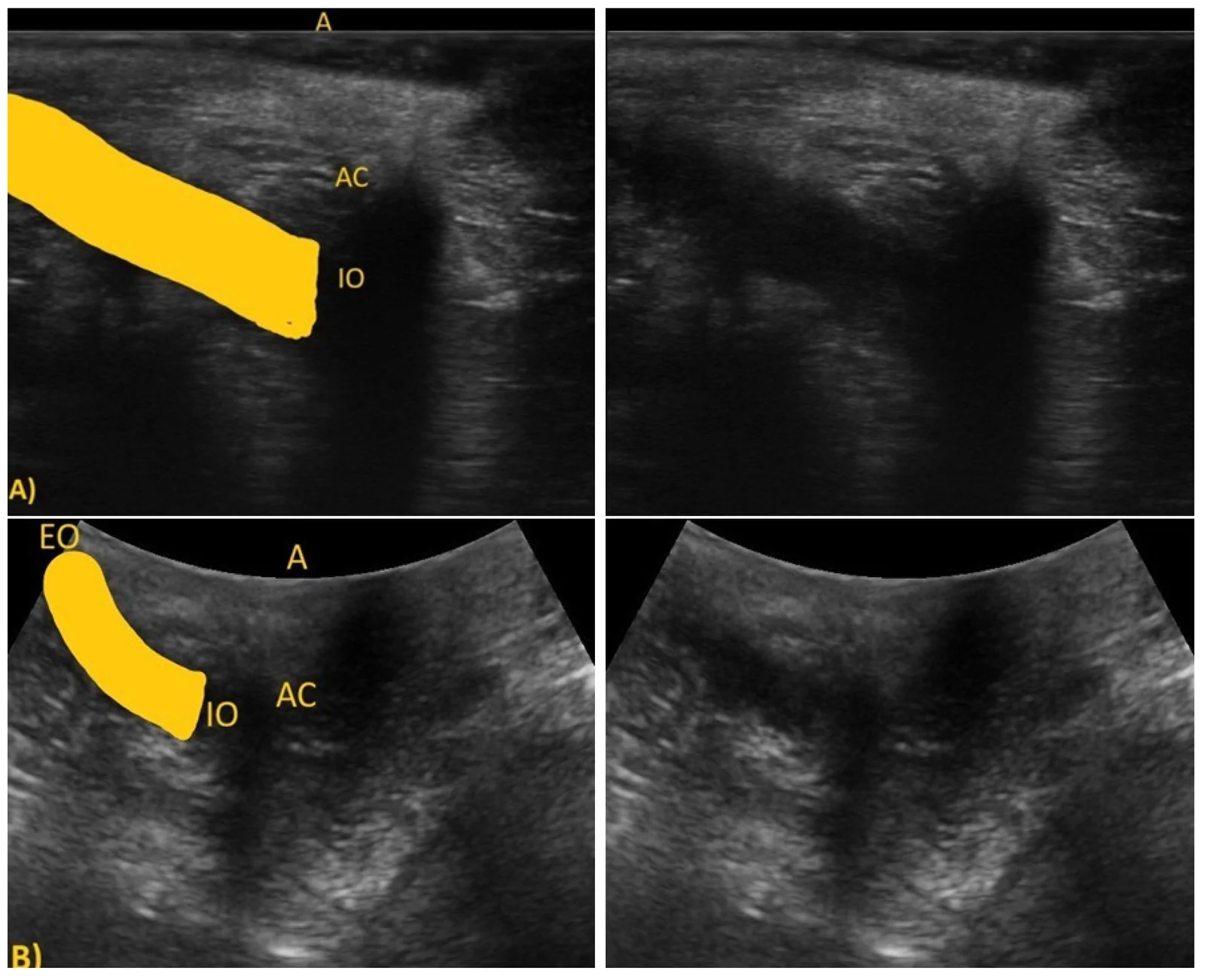
Ultrasound images: (**A**) were obtained using a superficial linear probe (7.5 MHz) and show a single low-type fistulous tract with obvious internal openings (IO); however, the length of the fistula makes it difficult to visualize the external opening (EO) on the same scan. (**B**) was taken with a deep curved probe (3.5 MHz) and shows the curved fistulous tract of 5 cm long with obvious internal openings (IO) into the anal canal (AC) located 21 mm away from the anal margin (A). The **left images** show the fistulous tract (thick yellow line), IO, and EO. The **right images** are corresponding unannotated scans.

**Figure 20:**
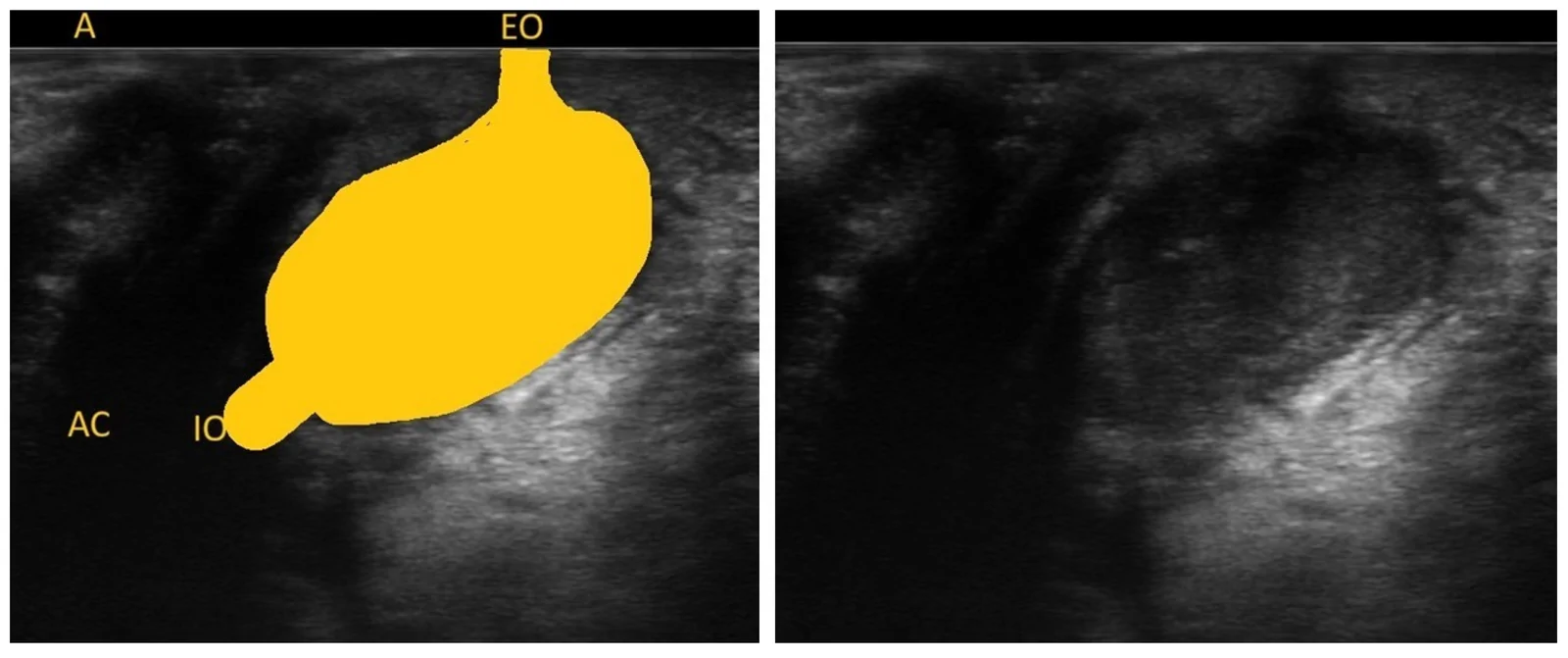
An ultrasound image obtained using a superficial linear probe (7.5 MHz) shows a right residual perianal abscess of 24 × 14 mm in maximal dimensions, with an obvious external opening (EO) and two internal openings (IO) into the anal canal (AC) located 19 mm and 16 mm from the anal margin (A). The other IO requires an additional scan to be observed. The **left image** shows the abscess (thick yellow line), EO, and IO. The **right image** is the corresponding unannotated scan.

In the lithotomy or left lateral decubitus position, TCUS is performed using a high-resolution 3–8 MHz linear probe or a 2–5 MHz sector probe applied percutaneously. The probe is covered with a latex sheath after applying contact gel. It is placed over the anal canal and used to scan the perianal region in the sagittal, coronal, and axial planes. TCUS can detect the IO, measure the length of the fistulous tract, and determine the distance of the IO from the anal margin. TCUS is a promising imaging modality for the diagnosis and classification of perianal fistulae [20]. When performed by an experienced operator, TCUS can effectively diagnose low perianal fistulae and assist in surgical planning. The advantages of TCUS include that it is non-invasive, well tolerated by patients, and free from ionizing radiation. Its main limitations are that it is operator-dependent and requires experienced sonographers and radiologists to interpret ultrasonography images [21]. TCUS is also limited by the requirement for modern ultrasound equipment and optimized acquisition settings to produce high-quality images and minimize interpretive pitfalls caused by gas and reverberation artifacts [22]; Figure 5, Figure 6, Figure 7, Figure 8, Figure 9, Figure 10, Figure 11, Figure 12, Figure 13, Figure 14, Figure 15, Figure 16, Figure 17, Figure 18, Figure 19 and Figure 20.

### 5.4. EAUS

EAUS was introduced by Law and Bartram in 1989. In the left lateral decubitus position, and without the need for anesthesia or specific patient preparation, EAUS is performed using high-resolution radial probes operating in the 6–16 MHz range. The probe is equipped with 360° mechanical rotation and provides a field of view (FOV) of up to 10 cm. EAUS is a practical, highly accurate, repeatable outpatient imaging technique for mapping perianal fistulae. It accurately identifies the IO, delineates the fistulous tract through the anal sphincters, and detects fistulous secondary branches and hidden abscesses. EAUS is safe, radiation-free, well tolerated, and can be performed at the bedside or intraoperatively for seton management. It is also a reliable alternative when MRI is contraindicated. However, EAUS has a limited FOV, which restricts the assessment of very lateral tracts, supralevator ani muscle tracts, and complex secondary branches existing beyond the FOV. Other limitations include its operator dependence and contraindication in patients with severe anal stenosis or acute anal pain that prevents probe insertion. Emerging modalities such as three-dimensional EAUS (3D-EAUS), contrast-enhanced ultrasonography, Doppler imaging, shear-wave elastography and artificial intelligence-assisted tract mapping may strengthen the role of EAUS [23]. 3D-EAUS has been demonstrated to effectively diagnose perianal fistulae [24].

### 5.5. MRI

MRI is the gold standard imaging modality for the evaluation of perianal fistulae and provides unmatched soft tissue visualization of the anal canal anatomy. It enables high-spatial-resolution mapping of the fistulous tract, accurate localization of the IO, assessment of involvement of the anal sphincters, identification of secondary tracts and abscesses, evaluation of supralevator collections, and detection of associated complications. MRI also facilitates serial evaluation, helping to guide treatment decisions and monitor treatment response. High-spatial-resolution, small FOV T2-weighted images are specifically useful for defining the relationship of the fistula to the anal sphincters, the intersphincteric plane and the levator ani muscle [25].

The European Society of Gastrointestinal and Abdominal Radiology (ESGAR) consensus guidelines recommend an MRI protocol that includes axial, coronal and sagittal T2-weighted sequences with or without fat suppression. Optional sequences include short tau inversion recovery (STIR), diffusion-weighted imaging, and fat-suppressed T1-weighted images after gadolinium administration. Both 1.5 T and 3 T MRI systems may be used. ESGAR recommends the use of a surface coil to enhance image quality and does not recommend endorectal receiver coils, as they are unnecessary for achieving adequate image quality. ESGAR also does not recommend routine patient fasting, rectal emptying or distension, the use of an anal marker or intravenous smooth muscle relaxants before imaging, and no special modifications are required for imaging children [26]. STIR sequences can demonstrate pus and granulation tissue without the administration of contrast media [27].

**Figure 21:**
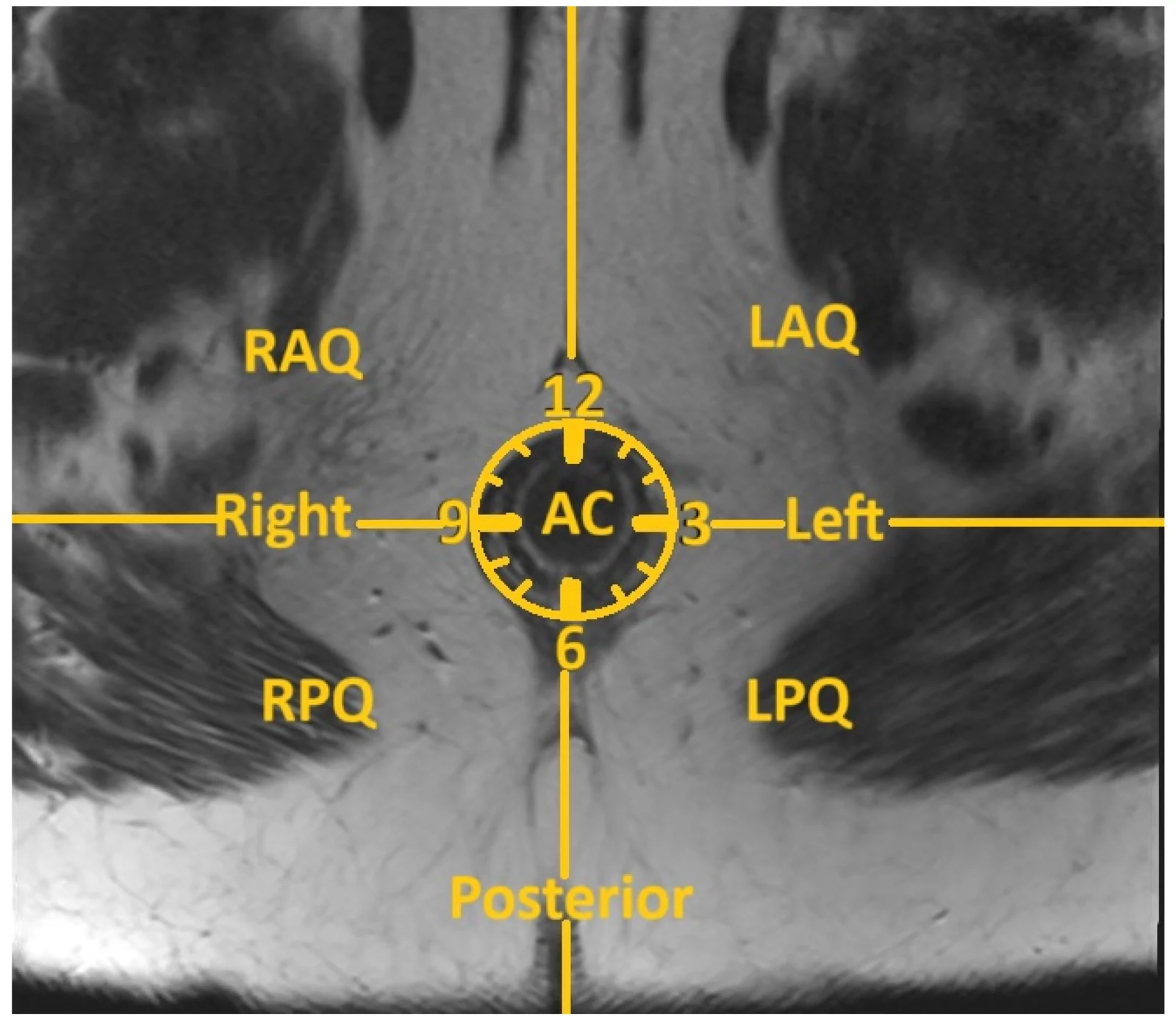
Axial magnetic resonance image of the perianal region in the lithotomy position, dividing the clockwise orientation of the anal canal into four quadrants and the 12 o’clock position. LAQ: Left anterior quadrant; LPQ: Left posterior quadrant; RPQ: Right posterior quadrant; RAQ: Right anterior quadrant; AC: Anal canal.

**Figure 22:**
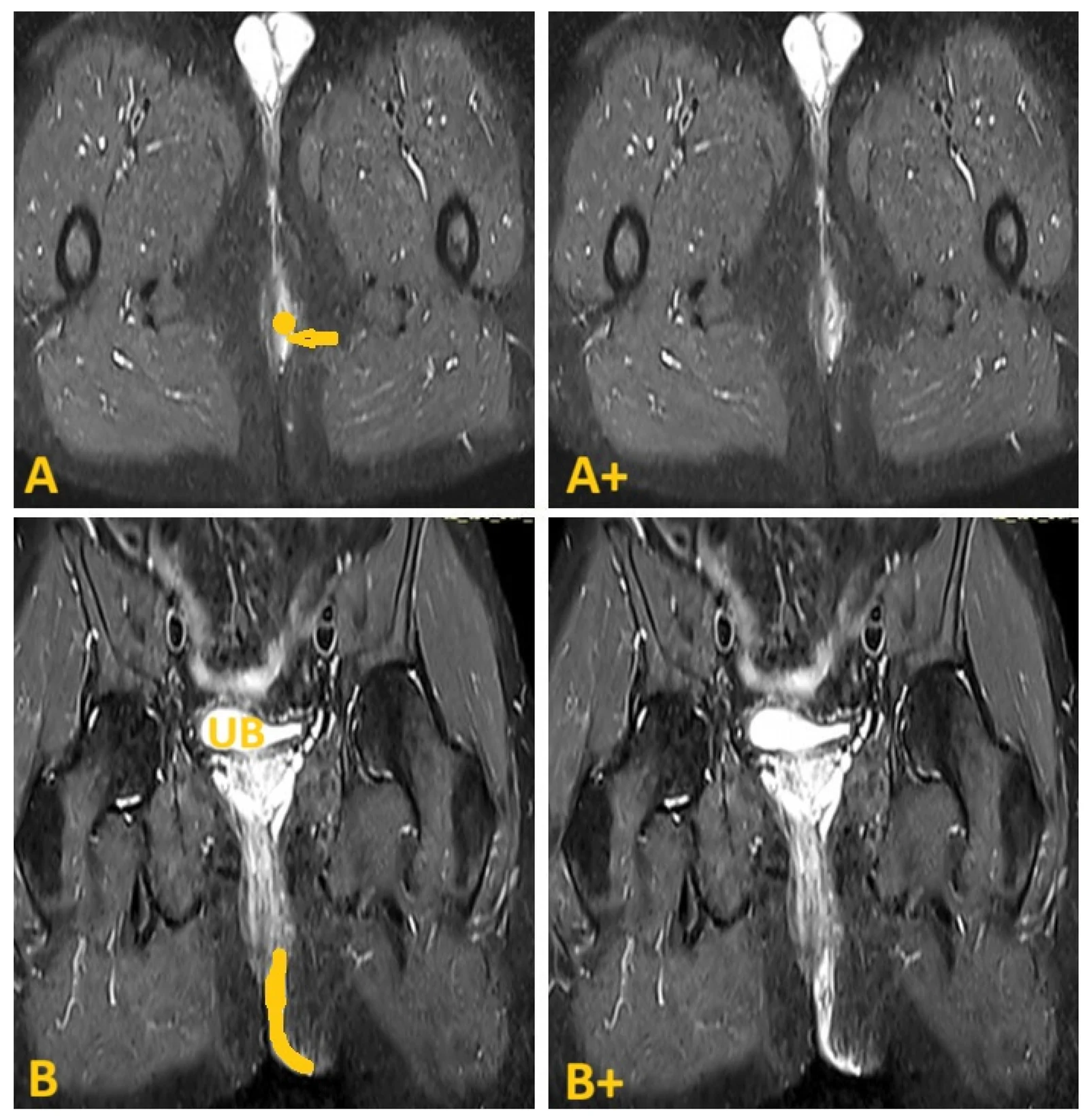
Selected magnetic resonance imaging images. (**A**) Axial STIR T2-weighted image showing a perianal fistula arising from the left posterior anal sphincter region at the 5 o’clock position (arrow) around the anal canal (dot) in the lithotomy position, with the internal opening located 20 mm from the anal verge. (**A+**) Corresponding unannotated image. (**B**) Coronal STIR T2-weighted image showing an intersphincteric fistulous tract measuring 53 mm in length and 8 mm in width, extending inferiorly in the left posterior quadrant to form an external opening. No branching or abscess formation is identified. (**B+**) Corresponding unannotated image. UB: Urinary bladder.

**Figure 23:**
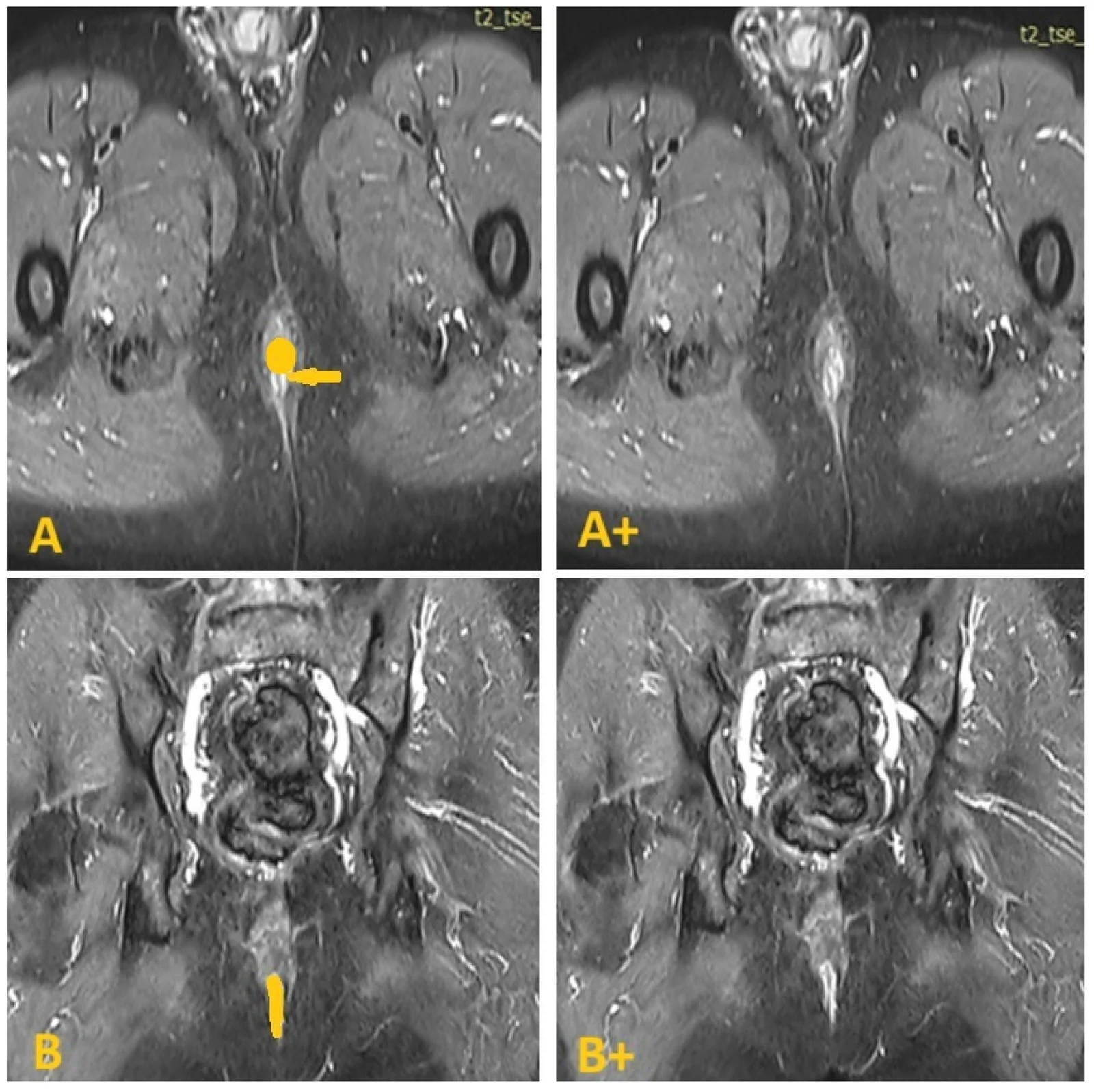
Selected magnetic resonance imaging images. (**A**) Axial STIR T2-weighted image showing a perianal fistula arising from the posterior anal sphincter region at the 6 o’clock position (arrow) around the anal canal (dot) in the lithotomy position, with the internal opening located approximately 20 mm from the anal verge. (**A+**) Corresponding unannotated image. (**B**) Coronal STIR T2-weighted image showing an intersphincteric fistulous tract measuring 35 mm in length and 3 mm in width, extending inferiorly in the posterior midline to form an external opening. No branching or abscess formation is identified. (**B+**) Corresponding unannotated image.

**Figure 24:**
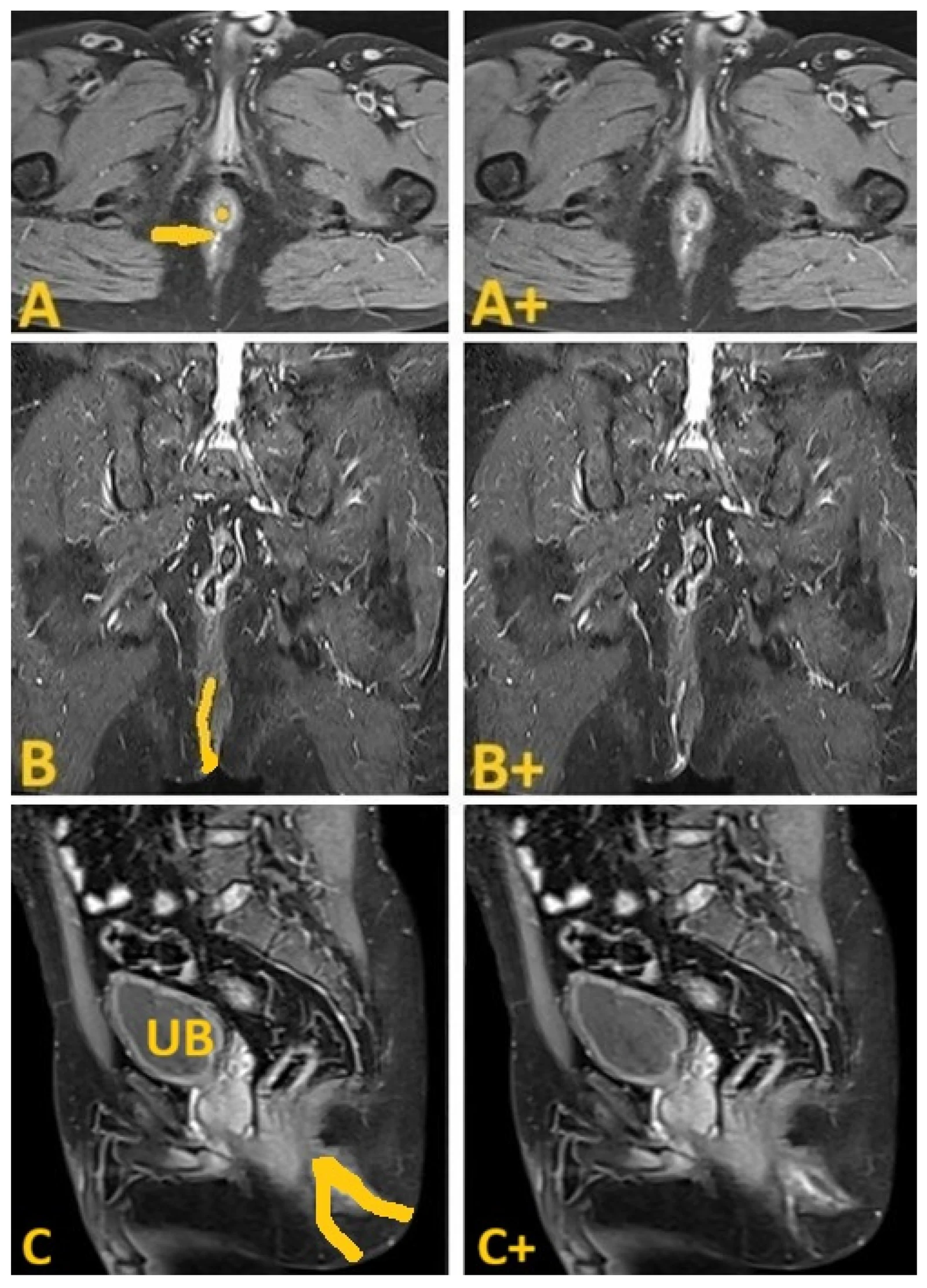
Selected magnetic resonance imaging images. (**A**) Axial DIXON T1-weighted image showing a perianal fistula arising from the right posterior anal sphincter region at the 7 o’clock position (arrow) around the anal canal (dot) in the lithotomy position, with the internal opening located approximately 35 mm from the anal verge. (**A+**) Corresponding unannotated image. (**B**) Coronal STIR T2-weighted image showing an intersphincteric fistulous tract measuring 60 mm in length and 3 mm in width, extending inferiorly in the right posterior quadrant to form two external openings. No abscess formation is identified. (**B+**) Corresponding unannotated image. (**C**) Sagittal STIR T2-weighted image showing part of the branching fistulous tract and the two external openings. (**C+**) Corresponding unannotated image. UB: Urinary bladder.

**Figure 25:**
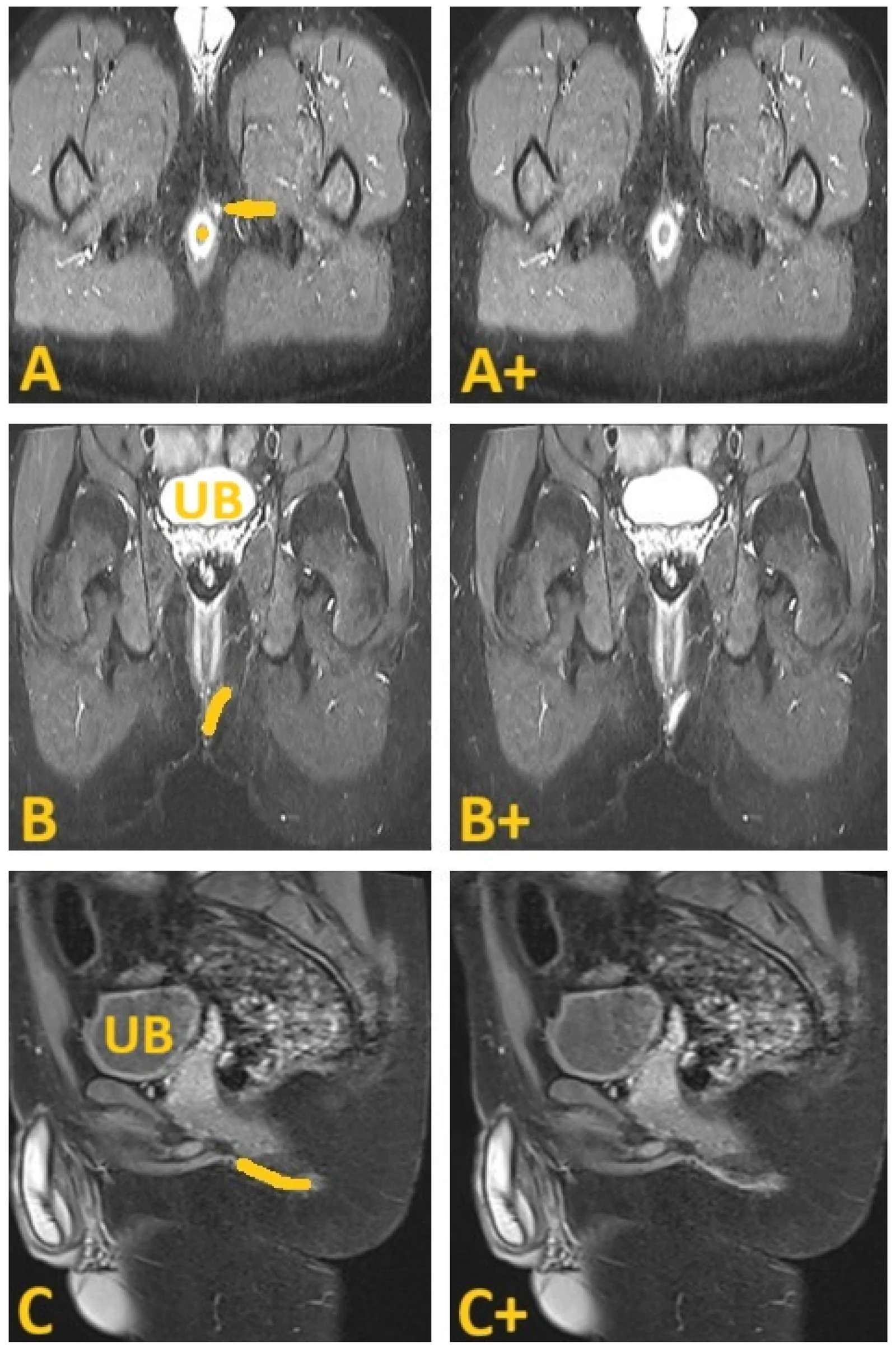
Selected magnetic resonance imaging images. (**A**) Axial STIR T2-weighted image showing a transsphincteric perianal fistula arising from the right anterior anal sphincter region at the 2 o’clock position (arrow) around the anal canal (dot) in the lithotomy position, with the internal opening located approximately 40 mm from the anal verge. (**A+**) Corresponding unannotated image. (**B**) Coronal STIR T2-weighted image showing the distal part of the fistulous tract, measuring 70 mm in length and 6 mm in width, extending inferiorly in the right anterior quadrant to form a single external opening. No branching or abscess formation is identified. (**B+**) Corresponding unannotated image. (**C**) Sagittal STIR T2-weighted image showing part of the fistulous tract. (**C+**) Corresponding unannotated image. UB: Urinary bladder.

**Figure 26:**
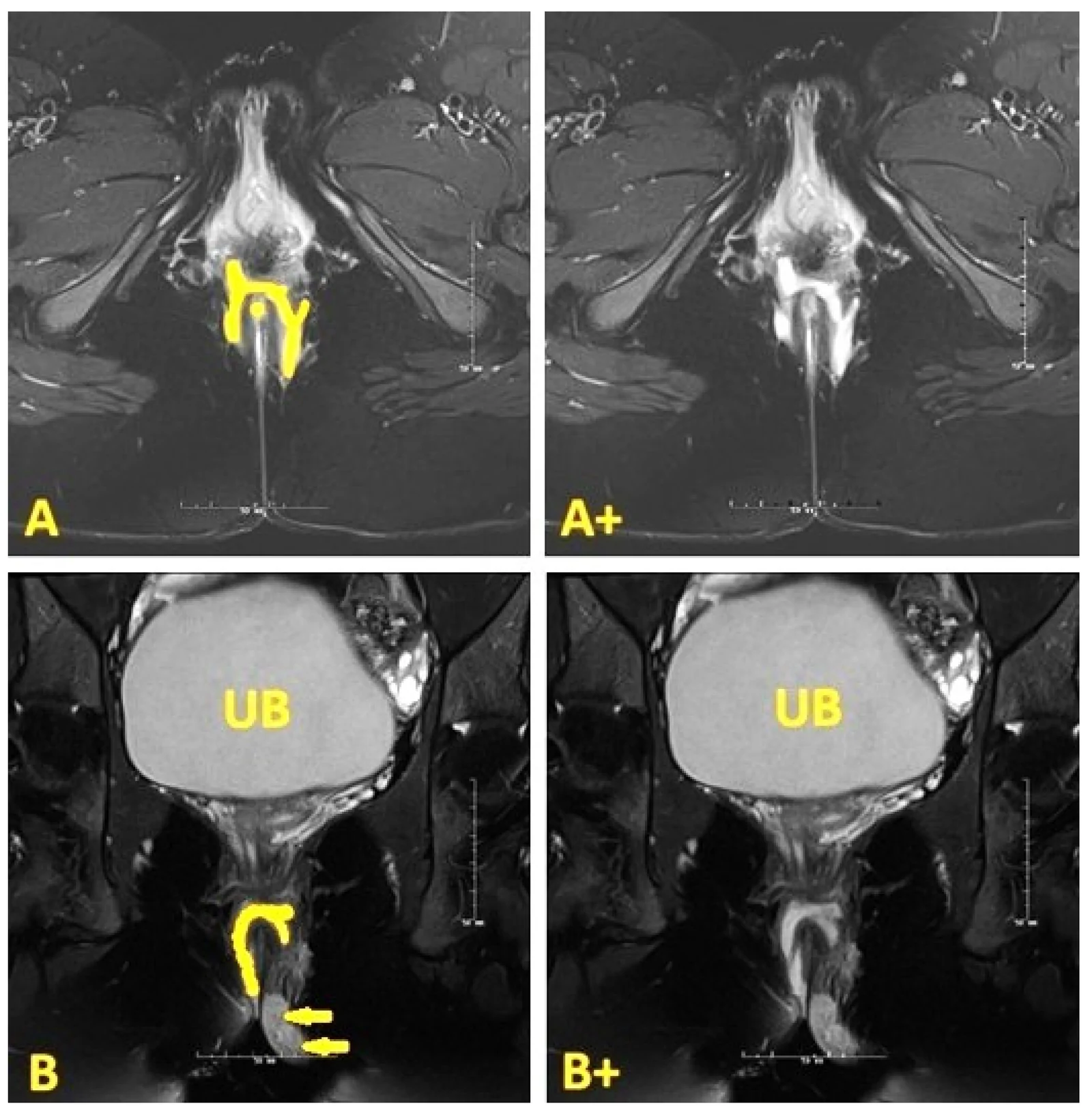
Selected magnetic resonance images of a patient with Crohn’s disease. (**A**) Axial fat-suppressed T2-weighted image showing a complex transsphincteric perianal fistula arising from the anterior midline anal sphincter region at the 12 o’clock position and extending into both sides of the perineum around the anal canal (dot), forming a horseshoe fistula. (**A+**) Corresponding unannotated image. (**B**) Coronal fat-suppressed T2-weighted image showing the complex fistula extending into both sides of the perineum, with greater involvement on the left side than on the right. Marked skin thickening and a hyperintense area are present in the left medial buttock, consistent with cellulitis (arrows). (**B+**) Corresponding unannotated image. UB: Urinary bladder.

**Figure 27:**
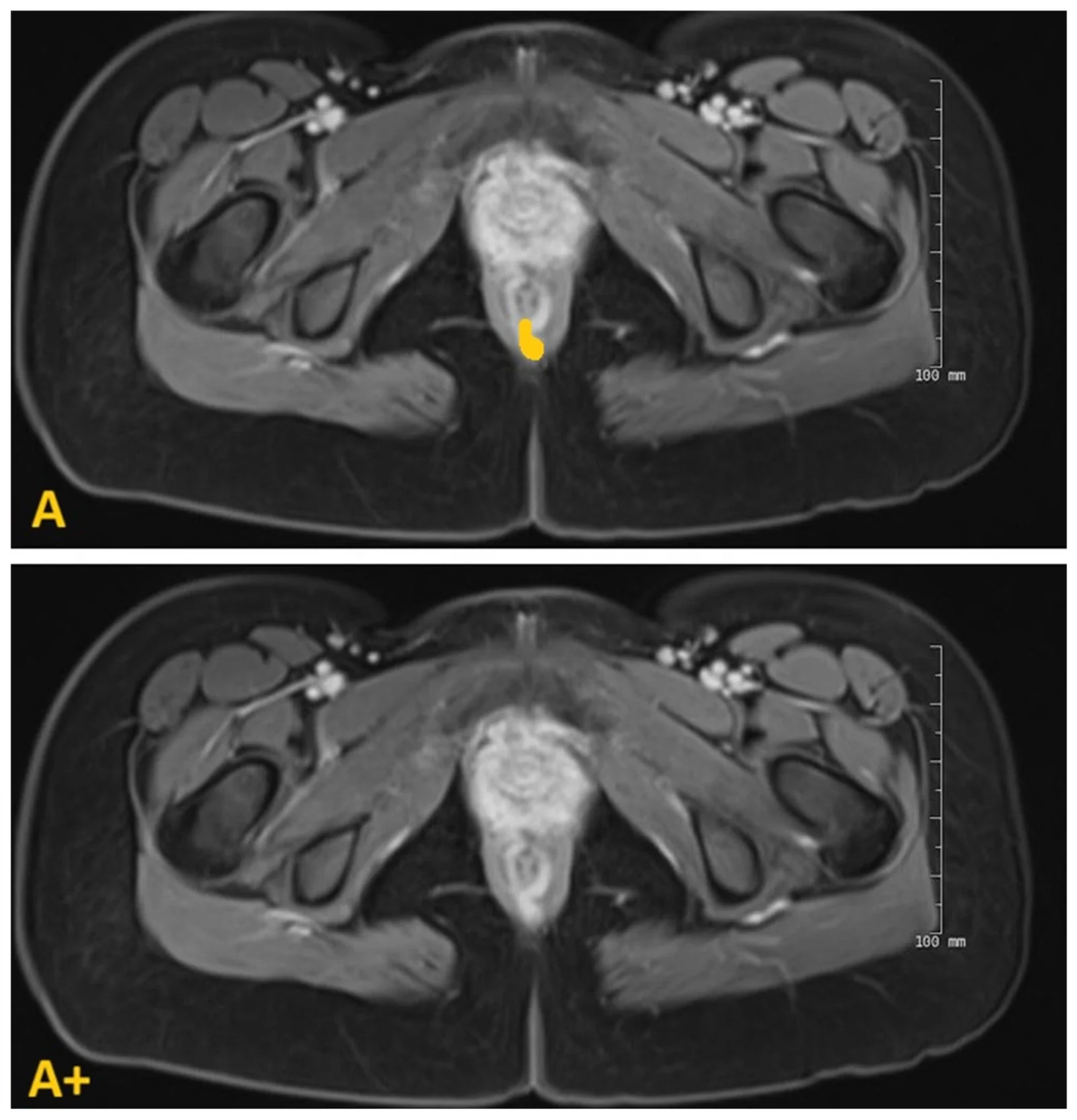
Selected magnetic resonance images of another patient with Crohn’s disease shown in Figure 26. (**A**) Axial post-contrast T1-weighted image showing a small blind-ending sinus tract (filled with yellow color) arising from the posterior midline around the anal canal. (**A+**) Corresponding unannotated image.

**Figure 28:**
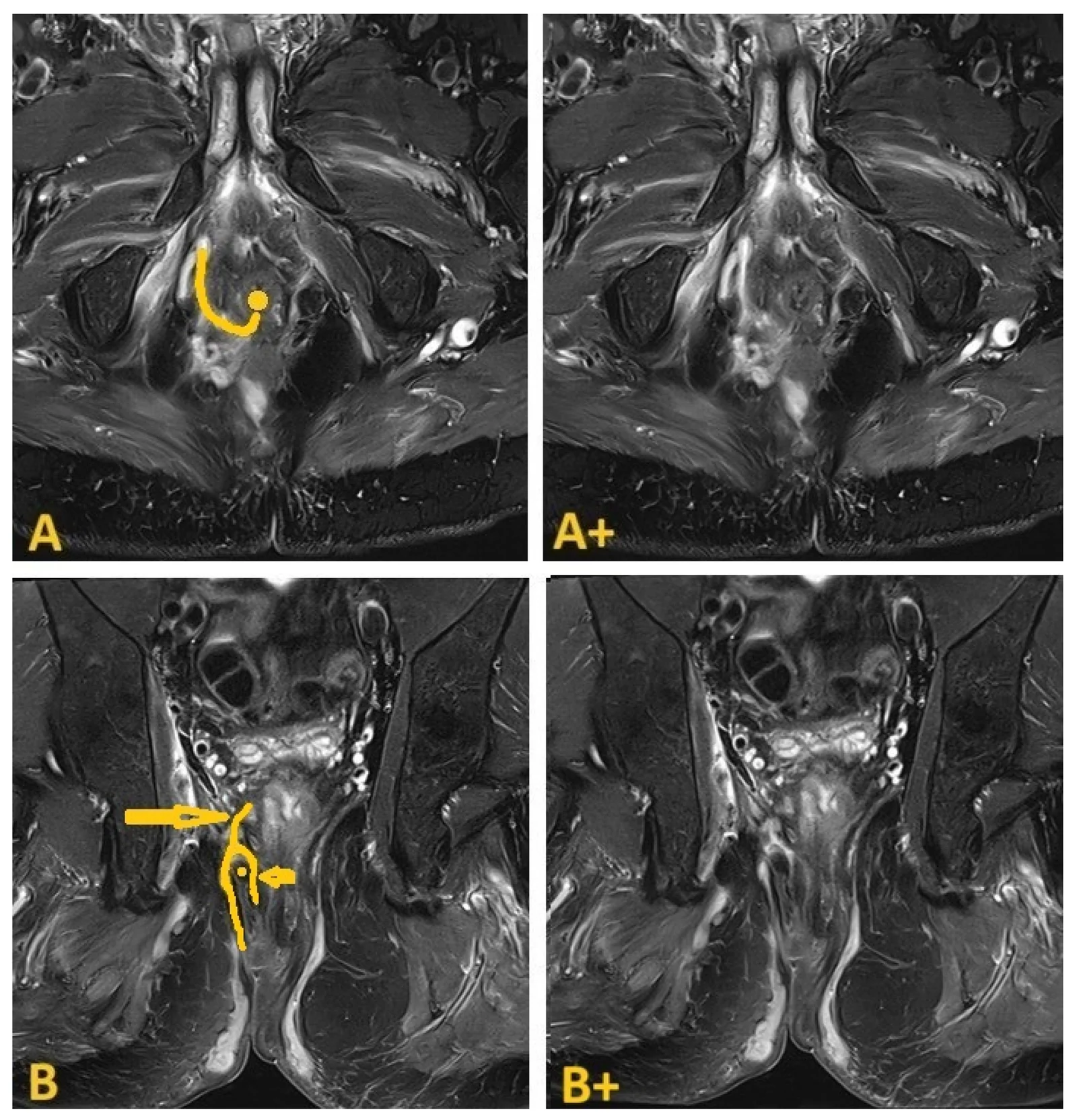
Selected magnetic resonance images. (**A**) Axial fat-suppressed T2-weighted image showing the internal opening of a perianal fistula arising from the posterior midline anal sphincter region at the 6 o’clock position. (**A+**) Corresponding unannotated image. (**B**) Coronal fat-suppressed T2-weighted image showing a solitary fistulous tract extending superior to the puborectalis muscle (dot) before dividing into two branches. One branch forms a suprasphincteric tract (short arrow) coursing inferiorly towards the anal canal and opening at the 6 o’clock position, while the second branch extends superiorly through the levator ani muscle to form an extrasphincteric tract (long arrow) opening into the rectum. (**B+**) Corresponding unannotated image.

According to the ESGAR recommendations, MRI reports should include the clinical details, Parks’ classification of each identified fistula, the total number of fistulae, and a complete description of each fistula. The report should document the radial location of the IO and EO using the clock-face nomenclature or the corresponding anal quadrant, the height of the IO in relation to the anal verge, dentate line and puborectalis muscle, the presence of multiple IOs or multiple fistulous tracts, the length of the fistulous tract where measurable, the location and size of any fistulous extensions, including intersphincteric, ischioanal or supralevator extensions, and the presence of horseshoe extensions. Less common findings, such as proctitis and sacral osteomyelitis, should also be reported. The report should comment on the integrity of the anal sphincters and include a comparison with previous MRI examinations, where available [26]. In our collected cases, all perianal fistulae were reported according to the ESGAR recommendations (Figure 21, Figure 22, Figure 23, Figure 24, Figure 25, Figure 26, Figure 27 and Figure 28).

Table 1 summarises the advantages and strengths of each imaging modality in diagnosing perianal fistulae, in addition to the weaknesses and limitations of each modality.

Finally, the successful use of a coring-out fistulectomy to treat perianal fistulae with secondary extensions while preserving the anal sphincters, even without pre-operative imaging, should be acknowledged, particularly in resource-constrained settings. Coring-out fistulectomy is a practical sphincter-preserving procedure that enables precise resection of the primary and secondary perianal fistulous tracts through intraoperative visual inspection and palpation aided by hydrogen peroxide injection. This procedure has been associated with high healing rates [37].

## 6. Limitations

This review focuses on TCUS and MRI, which are the most commonly used imaging modalities in Yemen. EAUS is not available in our clinical practice, which limits the generalizability of the findings to regions where this technique is routinely used. In addition, CT fistulography images were not included because of their infrequent use in our region. Diagnosis of perianal fistula is still difficult and requires highly experienced radiologists. Future research about shear-wave elastography and artificial intelligence-assisted tract mapping is recommended to introduce imaging modalities to facilitate perianal fistulae diagnosis.

## 7. Strengths

A major strength of this review is the inclusion of high-quality educational MRI images. As the most accurate imaging modality, MRI provides detailed visualization of the anal and perianal anatomy. The selected images also illustrate a wide range of presentations of perianal fistulae, supporting clear discussion of their classifications and relationships with surrounding structures.

The review is further strengthened by its extensive collection of TCUS images, which demonstrate the value of this modality in diagnosing perianal fistulae. This is particularly relevant in developing countries, where TCUS is widely available but access to 1.5 Tesla MRI systems may be limited.

## 8. Conclusions

In the imaging evaluation of perianal fistulae, conventional X-ray fistulography has become largely obsolete. Ultrasonography, including TCUS and EAUS, is widely available, radiation-free, and suitable for bedside use. Although effective in evaluating perianal fistulae, these techniques are operator-dependent and have limited FOV, which restricts their value in assessing high or complex perianal fistulae. In addition, EAUS may not be feasible in patients with severe anal stenosis or acute anal pain. CT fistulography remains an alternative for evaluating perianal abscesses when MRI and EAUS are unavailable or contraindicated.

Pelvic MRI remains the gold-standard imaging modality for evaluating perianal fistulae. Its superior soft tissue contrast enables detailed assessment of the anal canal anatomy, accurate localization of the internal opening, delineation of the fistulous tract in relation to the internal and external anal sphincters, and identification of secondary tract abscesses, and detection of associated complications. Consequently, MRI plays a central role in treatment planning and the assessment of treatment response.

## Figures and Tables

**Table 1:** Advantages and limitations of each imaging modality in diagnosing perianal fistulae.

Imaging Modality	Advantages and Strengths	Disadvantages and Limitations	Reference
X-ray fistulography	Can provide a quick, minimally invasive outpatient method to primarily outline perianal fistulae.	1—Poor diagnostic accuracy. 2—Poor soft tissue contrast prevents evaluation of the anal sphincters. 3—Fails to detect secondary hidden branches, extensions and abscesses adequately.	[18]
CT-fistulography	1—Wider availability and faster scan time than MRI. 2—Obvious visualization of contrast-filled fistulous tracts and abscesses. 3—Safe alternative when MRI is unavailable or contraindicated. 4—Multiplanar views to review anatomy in different sections.	1—Lower diagnostic accuracy than MRI. 2—Poor soft tissue contrast cannot differentiate fistulous tract, fibrosis and pelvic floor. 3—Cannot reliably outline the anal sphincters and levator ani muscles. 4—Incomplete contrast filling may omit diagnosis of secondary tracts or deep cavities. 5—Hazards of radiation exposure and contrast agents’ reactions.	[28,29,30]
Transcutaneous ultrasonography (TCUS)	1—Highly sensitive for detecting perianal fistulae and abscesses. It has 92% sensitivity and 97% specificity to detect fistulous tract complexity and the internal opening in comparison with the intraoperative findings. 2—Noninvasive and well-tolerated. 3—Repeatable at the bedside and in outpatient clinic. 4—Radiation-free. 5—Widely available. 6—Cost-effective.	1—Operator-dependent. 2—Low specificity to differentiate active fibrosis from granulation tissue. 3—Difficult to obviously map high or complex suprasphincteric or extrasphincteric extensions of perianal fistulae.	[31,32]
Endoanal ultrasonography (EAUS)	1—High sensitivity (92.2%) and specificity (100%) with 93.4% diagnostic accuracy for the diagnosis of perianal fistulae. EAUS has 94.6% accuracy for identifying the internal openings, and 87.4% diagnostic accuracy for determining fistula type according to the Park’s classification in comparison with operative results. 2—Provides high-resolution imaging with clear delineation of the anal sphincters and the intersphincteric plane. 3—Repeatable at the bedside and in outpatient clinic. 4—Radiation-free. 5—Widely available. 6—Cost-effective.	1—Highly operator-dependent. 2—Less effective for mapping high or complex suprasphincteric or extrasphincteric extensions of perianal fistulae. 3—Causes severe pain in patients with acute perianal sepsis. 4—Cannot pass through tight anal stenosis.	[23,33,34]
Pelvic magnetic resonance imaging (MRI)	1—Highly accurate with 97.5% diagnostic accuracy for the primary fistulous tract, 95.1% for secondary tracts, 92.6% for abscesses, 98% for horseshoe extensions, and overall accuracies ranging from 91.4% to 98.6%. 2—Providing exceptional soft tissue contrast with clear mapping of the primary tracts, secondary branches, and extensions relative to the anal sphincters. 3—Clearly identifies hidden fluid collections and active inflammation. 4—Can identify the internal openings and type of fistulous extensions. 5—Providing a roadmap for surgeons that reduces recurrence rate of fistulae.	1—Tiny secondary branches and tiny extensions may be missed. 2—Pinpointing the internal opening and differentiating active fluid-filled infection from inactive fibrosis or granulation tissue remains difficult in some patients. 3—Might be contraindicated in patients with non-MRI-compatible cardiac pacemakers, some metallic implants, and severe claustrophobia. 4—High cost and limited availability.	[19,35,36]

## Data Availability

No new data were created or analyzed in this study. Data sharing is not applicable to this article.

## Abbreviations

The following abbreviations are used in this manuscript:

CT	Computed tomography
MRI	Magnetic resonance imaging
TCUS	Transcutaneous ultrasonography
EAUS	Endoanal ultrasonography
FOV	Field of view
IO	Internal opening
EO	External opening
ESGAR	European Society of Gastrointestinal and Abdominal Radiology
STIR	Short T1-inversion recovery
